# Gastropod shell size and architecture influence the applicability of methods used to estimate internal volume

**DOI:** 10.1038/s41598-017-18906-6

**Published:** 2018-01-11

**Authors:** Marilia Nagata Ragagnin, Daniel Gorman, Ian Donald McCarthy, Bruno Sampaio Sant’Anna, Cláudio Campi de Castro, Alexander Turra

**Affiliations:** 1 Instituto Oceanográfico, Universidade de São Paulo (USP), São Paulo, SP Brazil; 2 School of Ocean Sciences, Bangor University, Menai Bridge, Anglesey, United Kingdom; 3 Instituto de Ciências Exatas e Tecnologia, Universidade Federal do Amazonas (UFAM), Itacoatiara, AM Brazil; 4 Hospital Universitário, Universidade de São Paulo (USP), São Paulo, SP Brazil

## Abstract

Obtaining accurate and reproducible estimates of internal shell volume is a vital requirement for studies into the ecology of a range of shell-occupying organisms, including hermit crabs. Shell internal volume is usually estimated by filling the shell cavity with water or sand, however, there has been no systematic assessment of the reliability of these methods and moreover no comparison with modern alternatives, e.g., computed tomography (CT). This study undertakes the first assessment of the measurement reproducibility of three contrasting approaches across a spectrum of shell architectures and sizes. While our results suggested a certain level of variability inherent for all methods, we conclude that a single measure using sand/water is likely to be sufficient for the majority of studies. However, care must be taken as precision may decline with increasing shell size and structural complexity. CT provided less variation between repeat measures but volume estimates were consistently lower compared to sand/water and will need methodological improvements before it can be used as an alternative. CT indicated volume may be also underestimated using sand/water due to the presence of air spaces visible in filled shells scanned by CT. Lastly, we encourage authors to clearly describe how volume estimates were obtained.

## Introduction

The evolutionary success of hermit crabs is closely linked to their habit of occupying empty gastropod shells, which need to be constantly upgraded to larger sizes as individuals’ grow^1^. Several parameters are known to influence the shell selection behavior of hermit crabs, including shell weight^2^, morphology^3^, density^4^ and internal volume^5,6^. Maintaining sufficient shell volume is essential; not only to permit growth, but also to provide sufficient refuge from predation^7^, desiccation, and thermal and osmotic stress^8,9^. Hermit crabs inhabit dynamic environments and have evolved to utilize a range of shell types, both between and within species^10^. Such plasticity in resource use can confound estimates of morphometric parameters, since crabs may inhabit shells that differ dramatically in terms of their size and architectural structure^11^. Shell type affects the growth rate of hermit crabs and heavy shells with a small internal volume will induce slower growth than lighter shells with a larger volume^12^. However, of all the traits affected by shell volume, its influence on reproductive success through the provision of brooding space for berried females (i.e., carrying eggs) may be the most beneficial^13^. Thus, given the pivotal role that shell volume plays in hermit crab biology and ecology, accurate measures of shell volume are crucial.

Internal volume has traditionally been estimated by filling the shell cavity with sand^13–24^ or water^12,25–28^. However, most studies reporting shell volume do not provide sufficient details on the methods used or whether estimates were derived from single or replicate measures, with the exception of Fotheringham^13^ who took 10 repeated measures of shell volume, but did not quantify precision. Similarly, given the techniques used to estimate volume, measurement inconsistencies may arise if the shell spire is not completely filled (i.e., when air spaces remain or the aperture is not uniformly filled to the same level). All of these aspects may increase variability in volume estimates that can hamper interpretations both within and across different studies. Thus, the level of variability that may be encountered when estimating shell volume needs to be quantified via replicate measurements made on the same shells using alternative methods. Given the enormous range of shell sizes and shapes (i.e., architecture) utilized by hermit crabs^11^, it is also important to understand how these factors may influence the accuracy of volume estimates.

In addition to the existing sand and water methodologies for estimating shell volume, newly available approaches such as Computed Tomography (CT) may offer a more accurate alternative for measuring shell internal volume. CT projects X-rays through an object of study, enabling a digital image reconstruction from profile slices^29^ to create a 3D representation of features such as a body part and its internal structures^30^. The technique has been gaining popularity across a wide range of biological and ecological fields^29,31–34^ and it may offer an alternative approach for measuring the internal volume of gastropod shells.

Thus, the aims of this study were: (1) to compare estimates of internal shell volume derived from three alternative methods (sand, water and CT) for five gastropod species that span a range of shell architectures (i.e., high-spired, medium-spired and low-spired shells) and sizes and; (2) to evaluate the reproducibility (expressed as Coefficient of Variation [CV] and Intra-class Correlation values [ICC]) of repeated measurements of internal shell volume measured using all three approaches.

## Results

### Component A: Comparison of shell volume estimates from three methods

#### Approach 1. Effect of method and shell architecture on volume estimate

**Figure 1:**
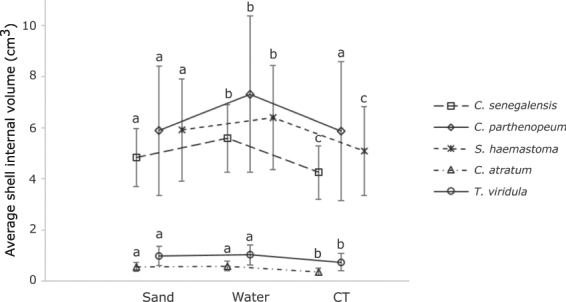
The average shell internal volume (Mean ± SD) estimated for five gastropod species of different shell architectures (n = 30 per species) using the three methods. The average volume derived from five replicate measures using sand and water methods and a single measurement using computed tomography (CT) (Approach 1). Different letters represent significant difference among methods for each shell species.

**Figure 2:**
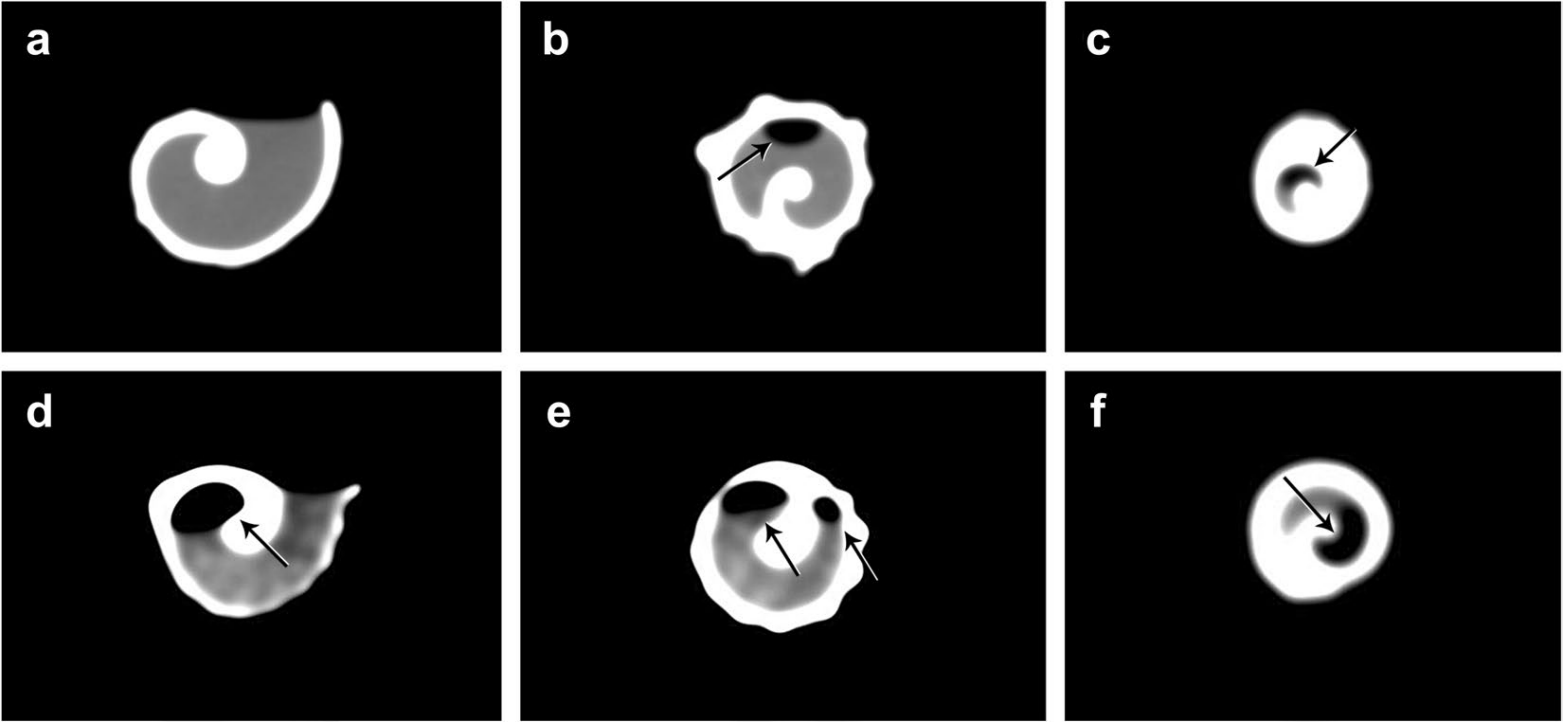
Computed Tomography slices of single gastropod shells filled with water (*Stramonita haemastoma*; (**a**) body whorl, (**b**) mid shell and (**c**) shell apex) and sand (*Cymatium parthenopeum*; (**d**) body whorl, (**e**) mid shell and (**f**) shell apex). The filled portion of the shell internal space is represented in gray, while the air spaces are represented in black (indicated by arrow). Note that the shell apex is not totally filled using either methods (**c**,**f**).

Variation in volume estimates was observed between methods [sand (S), water (W) and CT; Fig. 1 and Table 1]. The sand, water and CT methods gave significantly different shell volume estimates (repeated measures ANOVA, *F* = 791.94, *DF* = 2, *p* < 0.001) and there was a significant interaction between method and shell species (repeated measures ANOVA, *F* = 99.9, *DF* = 8, *p* < 0.001). The general pattern was for water to give higher estimates of shell volume compared to the other methods for the medium-spired species: *C. senegalensis* (Tukey test; W > S > CT), *C. parthenopeum* (Tukey test; W > S = CT) and *S. haemastoma* (Tukey test; W > S > CT) (Fig. 1). However, sand and water methods produced similar volume estimates, which were higher than the CT estimate for both high-spired (*C. atratum)* and low-spired (*T. viridula*) species [Tukey test; W = S > CT for both species]. Analysis of the CT results for shells filled with sand or water showed that both methods resulted in air spaces inside all shells scanned by CT, suggesting that these methods did not fill the shell cavity completely (Fig. 2).

**Table 1:** The effect of displacement method (Sand, S; Water, W) on measurements of internal shell volume (cm^3^) for five species of gastropod (n = 30 shells from the larger end of the size range for each species; Approach1). The variability in shell volume, based on five repeated measures of each shell, is expressed using the coefficient of variation (CV) and overall reproducibility represented by the intraclass correlation coefficient (ICC). *Note: all ICC values are significant at *p* < 0.001.

Shell	Method	Volume Average (range) – cm^3^	ICC (r)*	CV Average (range) – %
*Chicoreus senegalensis*	S	4.85 (3.18–7.69)	0.90	7.3 (2.2–11.7)
	W	5.59 (3.65–9.12)	0.97	3.8 (0.4–10.5)
*Cymatium parthenopeum*	S	5.88 (3.35–15.70)	0.96	8.6 (2.1–15.7)
	W	7.31 (3.90–18.27)	0.97	4.1 (0.9–11.5)
*Stramonita haemastoma*	S	5.91 (3.12–10.03)	0.98	4.9 (1.7–11.0)
	W	6.38 (3.48–10.16)	0.98	3.7 (1.2–15.5)
*Cerithium atratum*	S	0.57 (0.20–0.85)	0.76	14.0 (2.5–37.6)
	W	0.60 (0.24–0.99)	0.75	15.3 (3.9–29.6)
*Tegula viridula*	S	0.99 (0.48–2.09)	0.93	9.7 (2.5–30.2)
	W	1.03 (0.55–2.17)	0.94	9.1 (4.3–20.9)

#### Approach 2. Effect of shell architecture and size on volume estimate

**Figure 3:**
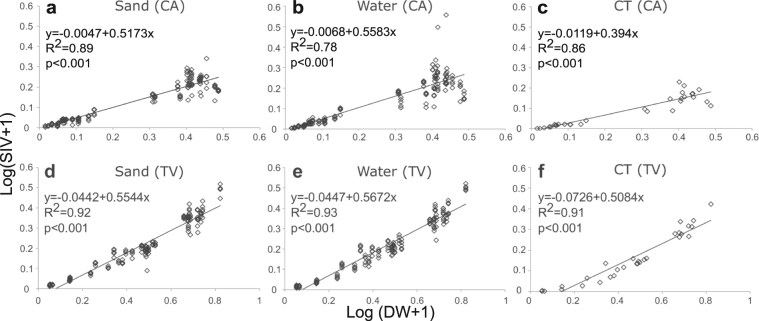
Relationship between shell dry weight (DW) and shell internal volume (SIV) estimates, using log(*x* + 1) transformed data, of 30 specimens of different sizes (Approach 2). (**a**) Sand, (**b**) water and (**c**) computed tomography (CT) methods for the high-spired shell species *C. atratum* (CA) and; (**d**) sand, (**e**) water and (**f**) CT methods for the low-spired shell species *T. viridula* (TV) respectively.

Regression analysis showed significant relationships between shell dry weight and volume estimates using the three methods for both *C. atratum* (Fig. 3a–c) and *T. viridula* (Fig. 3d–f). In both species, there was greater variability in volume estimates observed in large shells compared to small shells using all three methods. *Tegula viridula* showed stronger linear relationships for all three methods (r² > 0.91). Furthermore, the highest variability was observed in the volume estimates of large specimens of *C. atratum* due to the effects of both shell architecture and size.

### Component B: Examining the degree of reproducibility of shell volume estimates obtained using the three methods

#### Approach 3. Effect of method and shell architecture on reproducibility of volume estimate

Both sand and water produced significantly repeatable volume estimates for shells at the larger end of the size range for all five species (Table 1). Reproducibility, expressed by the ICC values (Table 1), was related to shell architecture and was higher (all r > 0.90 for both methods) for medium-spired shells (*C. parthenopeum, C. senegalensis* and *S. haemastoma*) and for the low-spired species (*T. viridula*) than for high-spired shell species (*C. atratum*, r = 0.76 for sand and r = 0.75 for water, respectively) (Table 1). The high-spired species *C. atratum* showed the highest average CV values for both methods, with CV of individual shells ranging between 2.5–37.6% using sand and 3.9–29.6% using water respectively (Table 1). In general, the low-spired (*T. viridula*) and medium-spired shells (*C. parthenopeum, C. senegalensis* and *S. haemastoma*) presented low average CV values for both methods (<10%; Table 1).

#### Approach 3. Interaction between shell architecture and shell size on reproducibility of volume estimate

Shell volume estimates using sand and water were also significantly repeatable for the two species with contrasting shell architecture, *C. atratum* and *T. viridula*, using the range of shell sizes available in nature (Table 2a). However, volume estimates were less reproducible for the high-spired *C. atratum* using water (r = 0.72) compared to sand (r > 0.90). In contrast, the low-spired *T. viridula* showed high reproducibility in volume estimates (r > 0.95) using both methods (Table 2a).

**Table 2:** The effect of displacement method (Sand, S; Water, W) on measurements of internal shell volume (cm^3^) for *Cerithium atratum* and *Tegula viridula* for (A) shells from the full size range found in nature for each species (n = 30) and (B) for size classes defined as ‘small’ and ‘large’ sized specimens (n = 15 per size class) (Approach 2). The variability in shell volume, based on five repeated measures of each shell, is expressed using the coefficient of variation (CV) and the overall reproducibility represented by the intraclass correlation coefficient (ICC). Note the significance values of p < 0.001** and p < 0.05* based on ANOVA^53^.

Shell		Method	Volume Average (range) – cm³	ICC (r)	CV Average (range) – %
(A)					
*Cerithium atratum*		S	0.37 (0.02–0.85)	0.94**	18.8 (2.5–55.1)
		W	0.38 (0.01–0.99)	0.72**	18.1 (3.9–55.4)
*Tegula viridula*		S	0.70 (0.03–2,09)	0.97**	10.0 (2.5–30.2)
		W	0.72 (0.02–2.17)	0.98**	11.5 (4.3–33.8)
(B)					
*Cerithium atratum*	small	S	0.10 (0.02–0.42)	0.94**	25.3 (7.5–55.1)
		W	0.11 (0.01–0.51)	0.98**	21.0 (3.9–55.4)
	large	S	0.64 (0.40–0.85)	0.65**	12.3 (2.5–29.9)
		W	0.71 (0.35–1.10)	0.27*	24.0 (6.3–77.2)
*Tegula viridula*	small	S	0.30 (0.03–0.60)	0.98**	9.3 (5.0–17.4)
		W	0.32 (0.02–0.66)	0.93**	15.0 (7.5–33.8)
	large	S	1.09 (0.48–2.09)	0.96**	10.6 (2.5–30.2)
		W	1.12 (0.55–2.17)	0.95**	8.1 (4.3–21.0)

When small and large shells were analysed separately, both the sand and water volume estimates showed high reproducibility for small shells of *Cerithium atratum* (r ≥ 0.94, Table 2b). However, volume estimates for large shells were less repeatable (Sand, r = 0.65; Water r = 0.27; Table 2b) using both methods, indicating the greatest variability in volume estimates for large individuals in high-spired shell species (Fig. 3a–c). For *Tegula viridula*, volume estimates were significantly reproducible for both size classes using both the sand and water methods (all r > 0.90; Table 2b).

#### Approach 4. Reproducibility of volume estimates using CT compared to sand and water methods

Volume estimates for shells at the larger end of the size range were significantly repeatable for all five shell species using all three methods, except for *C. atratum* using sand (Table 3). In general, the CT method demonstrated low variability in repeated estimates for all shell species, with CV values < 6.5% (Table 3). For the high- and low-spired species (*C. atratum* and *T. viridula*), CT presented the highest ICC values and the lowest CV values (Table 3). It should be noted that the reproducibility of sand and water methods is lower here compared to approach 3 due to the reduced sample size (n = 3 cf. n = 30 in approach 3), however, the aim of this analysis was to directly compare the pattern of reproducibility for CT when compared to the displacement methods.

**Table 3:** The effect of method (Sand, S; Water, W; Computed Tomography, CT) on measurements of internal shell volume (cm^3^) for large shells of the five gastropod species and for small specimens of *Cerithium atratum* and *Tegula viridula* (n = 3 for each group) (Approach 4). Variability in shell volume (based on 5 repeated measures) is expressed using the coefficient of variation (CV, %) and the overall reproducibility represented by the intraclass correlation coefficient (ICC) with associated p-value based on ANOVA^53^. NS = ICC value not calculated as ANOVA^53^ was non-significant.

Shell		Method	Average volume (range) – cm³	ICC (r)	*p*	CV Average (range) – %
*Chicoreus senegalensis*		S	5.24 (4.80–6.08)	0.76	<0.001	7.38 (4.90–11.68)
		W	5.91 (5.49–6.61)	0.85	<0.001	4.0 (2.85–11.68)
		CT	5.30 (4.80–5.96)	0.79	<0.001	4.32 (1.35–9.71)
*Cymatium parthenopeum*		S	7.02 (5.85–8.32)	0.84	<0.001	7.02 (7.96–9.38)
		W	8.49 (7.56–9.38)	0.66	0.002	8.49 (7.96–9.38)
		CT	7.89 (7.54–8.52)	0.86	<0.001	2.61 (0.98–3.46)
*Stramonita haemastoma*		S	7.09 (6.26–8.43)	0.98	<0.001	2.16 (1.66–3.09)
		W	7.70 (6.67–8.86)	0.68	0.002	7.70 (2.90–15.48)
		CT	7.21 (6.26–8.46)	0.84	<0.001	6.13 (2.90–8.45)
*Cerithium atratum*	small	S	0.11 (0.08–0.12)	0.67	0.002	11.90 (2.66–28.41)
		W	0.12 (0.08–0.15)	0.94	<0.001	6.57 (4.92–9.60)
		CT	0.07 (0.06–0.07)	0.23	0.03	8.31 (2.53–13.69)
	large	S	0.64 (0.54–0.80)	NS	0.55	20.95 (14.67–29.85)
		W	0.59 (0.48–0.71)	0.58	0.007	15.84 (8.22–26.56)
		CT	0.54 (0.46–0.64)	0.98	<0.001	1.94 (1.22–2.60)
*Tegula viridula*	small	S	0.22 (0.14–0.33)	0.97	<0.001	7.10 (5.35–10.36)
		W	0.23 (0.16–0.32)	0.93	<0.001	8.7 (4.72–12.78)
		CT	0.16 (0.10–0.24)	0.95	<0.001	7.94 (5.53–10.51)
	Large	S	1.21 (1.04–1.40)	0.64	0.003	10.98 (8.46–12.91)
		W	1.25 (1.07–1.46)	0.74	<0.001	8.9 (4.52–11.09)
		CT	1.17 (0.95–1.41)	0.87	<0.001	6.32 (3.27–8.9)

When the volume estimates derived from all three methods were compared for *C. atratum* and *T. viridula*, reproducibility was higher for small specimens than for large specimens. However, while the CT method showed low reproducibility (r = 0.23) for small shells of *C. atratum*, the same approach conversely showed the highest reproducibility for large specimens (r = 0.98). For this species, the water method yielded the highest reproducibility for small shells (r = 0.94). For *T. viridula*, all methods showed higher reproducibility for small shells (r > 0.90) than large shells (Table 3). Thus, the degree of reproducibility in volume estimates was related to shell architecture and size, but not always in a predictable way.

## Discussion

The use of standard methods for measuring biological units is vital for comparative studies across time and space^35–39^. For gastropods and other shell-inhabiting invertebrates such as hermit crabs, this is reflected in the need for accurate and reproducible ways of measuring shell volume to ensure consistency and comparability across studies. This study provides the first assessment of the precision and reproducibility of traditional displacement methods and investigates the potential for using computed tomography (CT) as an alternative approach for deriving shell volume estimates.

Repeated measures of volume varied not only according to the method used, but were also dependent on shell size and architecture. Although care was taken to ensure consistency when applying the sand and water methods, the observed variability in volume estimates probably relates to factors such as variation in the meniscus level for water, the degree of compaction for sand and the presence of air spaces within the shell when filled. The consistently lower volume estimates derived from CT were unexpected and may, in part, result from inconsistent application of clay, or be due to low sensitivity and/or inappropriate resolution or settings which may have hampered the distinction between internal air space and shell structure by the CT scanner. However, the use of CT did highlight the presence of airspaces providing a possible explanation for the observed variation in volume estimates using the sand and water methods and indicating that both methods may still underestimate the true internal volume of a gastropod shell.

Despite the inconsistencies inherent in the sand and water methods, our results suggest that for the majority of studies conducted on shells spanning a typical range of sizes and architectural types, a single volume displacement measurement is probably sufficient to derive ecological conclusions as ICC values were generally high (>0.90) and CV values were low (<15%) across methods and shell types (especially for medium spired shells). This result provides a general validation of the sand^13,14,19^ and water^12,25,26^ methods used in the majority of past studies examining gastropod shell volume- hermit crab relationships. However, although average CV values for displacement methods were generally low, shell CV values >30% were recorded for some high and low spiral pattern shells. Displacement methods were less repeatable for large shells than small shells in both low- and high-spired species and variability in volume estimates obtained for all methods increased with shell weight for both *C. atratum* and *T. viridula*. Hence, these results highlight the influence of size and architecture on the reproducibility of volume estimates and indicate a requirement for multiple repeated measures of volume for species with certain types of complex architecture.

The use of single volume estimates may be applicable for broad-scale studies of hermit crab ecology where a certain degree of error may be acceptable, e.g., Floeter *et al*.^25^ who showed a general relationship between selection and shell volume but not weight. However, replicate measures might be warranted where research questions are aimed at understanding finer-scale dynamics such as reproductive-growth trade-offs^14^, predation susceptibility^40^ and decisions about resource value^41^. In studies where accuracy and precision are highly desirable, careful consideration of method would be advisable given that estimates of volume depend on the material used (e.g., volume estimates obtained by water were typically higher than sand, with both potentially impacted by air spaces) and shell architecture (CV values are higher for high-spired than for low-spired species). Low reproducibility in volume estimates may occur as a consequence of the physical nature of the materials used (e.g., air present in bubbles in water and inter-grain air spaces in sand), or because of inconsistencies in defining when a shell is considered ‘full’ of sand or water. It is possible that inconsistencies could be minimized during specimen preparation by putting a few drops of ethanol into the shell to fully moisten the internal surface to make it more hydrophilic and subsequently removing the ethanol with a vigorous shaking before filling the shell with water (personal communication, Dr. A. Richard Palmer, University of Alberta). Although this approach was not applied in the present study, it could be tested in subsequent studies. In addition, CT offers the potential to give very precise volume estimates as our preliminary data indicated reproducibility was generally comparable or better for most shell types and sizes. However, it provided lower volume estimates compared to the displacement methods and will need further methodological development, validation and evaluation before it can be used as a realistic alternative to traditional displacement methodologies.

During the course of this study we discovered a general absence in the existing literature of detailed descriptions of the protocols and levels of replication employed for the sand and water methods (e.g., the rationale behind calculating sand volume from sand weight, how to minimize the risk of sand compaction, how to prevent water leaks and to define meniscus level). We suggest that where the objective of scientific research is to provide fine-scale contrasts in shell morphology (e.g., shell adequacy) the adoption of a protocol that includes replicate measures (for at least a subset of specimens) and presents measures of variance for statistical comparison may improve generality across studies. In general, using replicate measures may help to ensure confidence in the values estimated from traditional sand and water methods.

In conclusion, our results suggest that the traditional displacement methods commonly used to estimate shell volume (i.e., filling with sand and water) are generally appropriate for the majority of broader ecological studies and that a single measurement will typically suffice. However, care must be taken when using these methods on shells that differ in terms of size and/or shape, as error typically increases with size and spiral architecture, decreasing reproducibility. Overall, our observations highlight the need for researchers to be aware that all three methods yield variation in shell volume estimates, in terms of precision and accuracy that relate to shell characteristics. Regardless of the approach adopted, we encourage authors to clearly describe how volume was measured, including details on reproducibility (number of replicates taken). Similarly, we encourage ongoing tests of new methodologies as they become available, which might provide more accurate and precise estimates as demonstrated through high-resolution imaging of small animals^42–44^ and other specimens^42,43,45,46^ using micro-CT. Further, it presents comparatively higher spatial resolution^42^, which is described as the required distance between two adjacent structures of the study object to be distinguishable in the images captured by the equipment (i.e., a parameter related to the size of the voxel and thereby accuracy of image reconstruction)^47,48^. Thus, limitations of clinical CT scanners, such as spatial resolution^49^, may also have influenced the accuracy of shell volume estimates in the present study. Improving the precision of the methodological inferences upon which we build our knowledge, is not only likely to give us greater confidence in our own conclusions, but will almost certainly increase the capacity to cumulate data from different studies and across a range of spatial and temporal scales.

## Methods

### Shell species

**Figure 4:**
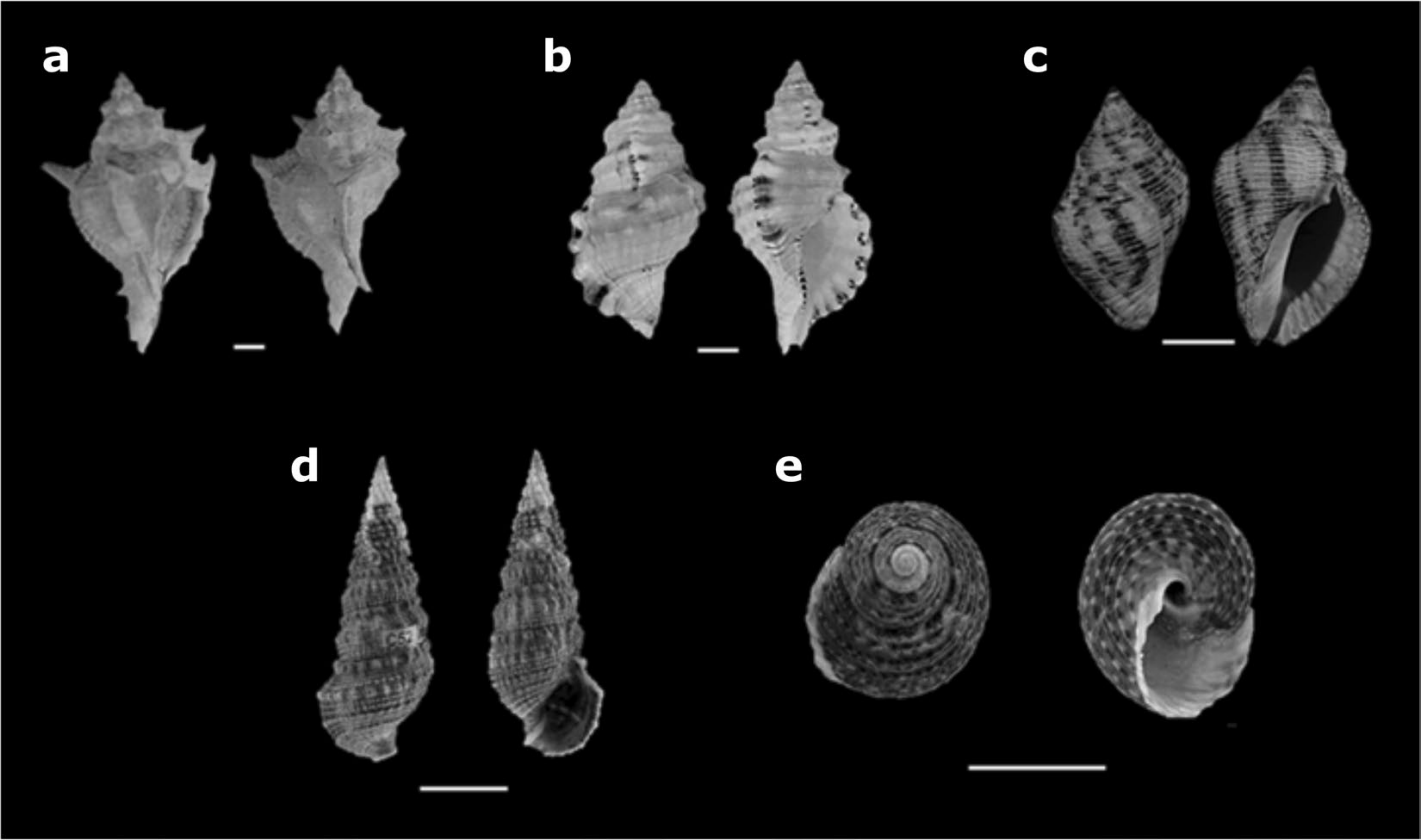
Gastropod species used to measure shell volume: (**a**) *Chicoreus senegalensis* (**b**) *Cymatium parthenopeum*, (**c**) *Stramonita haemastoma*, (**d**) *Cerithium atratum* and (**e**) *Tegula viridula*. These species represent (**a**–**c**) elongated/medium spired, (**d**) high-spired and (**e**) globose/low-spired shells respectively. Scale bar = 1 cm. Photographs of panels (a), (b) and (c) were taken by Ragagnin, M.N. and photographs from panels (d) and (e) were reprinted from Dominciano *et al*.^54^ with permission from Elsevier, under license number 243020641674.

We selected the shells of five gastropod species that are regularly used by intertidal hermits crabs^11,50,51^, but which vary in their overall size and architecture. The species included: the elongated/medium-spired *Chicoreus senegalensis* (Gmelin, 1790), *Cymatium parthenopeum* (Von Salis, 1793) and *Stramonita haemastoma* (Linnaeus, 1767); the high-spired *Cerithium atratum* (Born, 1778); and the globose/low-spired *Tegula viridula* (Gmelin, 1791) (Fig. 4). Variation in the shell weight and shape of these species has been previously described^12^. For each species, estimates of shell volume were derived for the same specimens using the sand, water and CT methods. For all specimens, the siphonal canal was covered by clay to prevent the escape of water or sand during volume estimates and to exclude the siphonal canal from the volume estimate.

### Estimates of shell volume

#### Sand

Shells that had been pre-weighed (dry weight, g) using an analytical balance (±0.00001 g) were filled with fine dry sand (grain size between 0.125 and 0.250 mm Ø) using a spatula that ensured sand was not forced into the shell to prevent variations in compaction. As the sand was added, the shell was held in a vertical position (shell apex downward) and tapped by hand to ensure complete penetration of the internal cavity. When the spire was fully filled and sand was visible at the beginning of body whorl, each shell was gently and slowly tilted to a horizontal position whilst more sand was added to fill the body whorl. The shell was deemed full once the aperture was completely filled with sand. Care was taken to ensure that the sand level did not exceed the upper edge of the shell aperture. Each shell was re-weighed after filling and the mass of sand (g) calculated as the difference in shell dry weight. To convert the mass to a volume, a 1 cm³ container was filled with sand to replicate the same procedure used for shells. To ensure the accuracy of this procedure, it was repeated five times, and the conversion factor was calculated as the mean of the five estimates (Mean ± SD = 1.687 ± 0.066 g), according to the equation v = m/1.687, where v is the shell volume (cm³) and m is the mass (g) of the sand within the shell. To check for the presence of air spaces or other irregularities (such as differences in compaction) within the shell, three sand-filled specimens of each shell species were examined using CT.

#### Water

Prior to measurements, industrial silicone was applied to the entire outer surface of each shell to prevent leakage through microscopic perforations. After coating with silicone, the shells were weighed and the shell cavity filled with distilled water using a pipette or syringe, depending on the shell size. Water was carefully added with the shell maintained in a vertical position (shell apex downward). Before the shell was completely full, the shell aperture was blocked using a finger or thumb and the shell was gently shaken to facilitate water penetration of the last spire. The shell was then slowly tilted to the horizontal position (aperture upward) whilst at the same time water was added until the body whorl was full. Each shell was considered full when the margin of the meniscus of the water reached the upper edge of shell aperture. The mass of the shell filled with water was then measured as above. As the density of distilled water is 1 g/cm³, the internal volume was obtained from the difference between the mass of the filled shell and the pre-weighed empty shell. To check for possible air spaces formed by the water method, three specimens of each shell species were filled with water and examined by CT as was done for sand.

To determine whether the silicone coating would absorb water and affect the shell weight measurements, ten shells coated with industrial silicone were randomly selected, placed in an oven (60 °C for 12 h) and the dry weight obtained immediately after the shell was removed from the oven. After a few minutes, the shells were re-weighed to observe possible variations in dry weight caused by the industrial silicone absorbing moisture from the air. This procedural control showed that the use of silicone did not affect the dry weight (paired *t* = −1.001; *DF* = 9; *P* = 0.34) and therefore the final calculation of volume for the water method.

#### Computed Tomography

To standardize this method and define an “internal space”, the shell aperture was sealed with a thin layer of clay to isolate the air inside the shell from the outside environment. This procedure was performed without pressing the clay inside the aperture to avoid any influence on the volume estimates. This enabled quantification of the volume of air inside the cavity, which gives the total internal volume of the shell.

The type of CT technique employed was ‘multi-slice’ tomography, using a Philips Brilliance CT 64-channel scanner (Philips Medical Systems, Amsterdam, The Netherlands) to capture the images. The information system coupled to the scanner (Philips CT Viewer software) was used to manipulate the image data and derive the volume estimates. The scan parameters were set at: 120 kV, 100 mA/slice, 0.5 s of rotation time, collimation of 64 × 0.625 mm, 512 × 512 matrix size, 54 mm field of view (FOV), pitch factor of 0.891, standard filter, standard resolution, slice thickness of 0.67 mm with 0.33 mm of increment.

**Figure 5:**
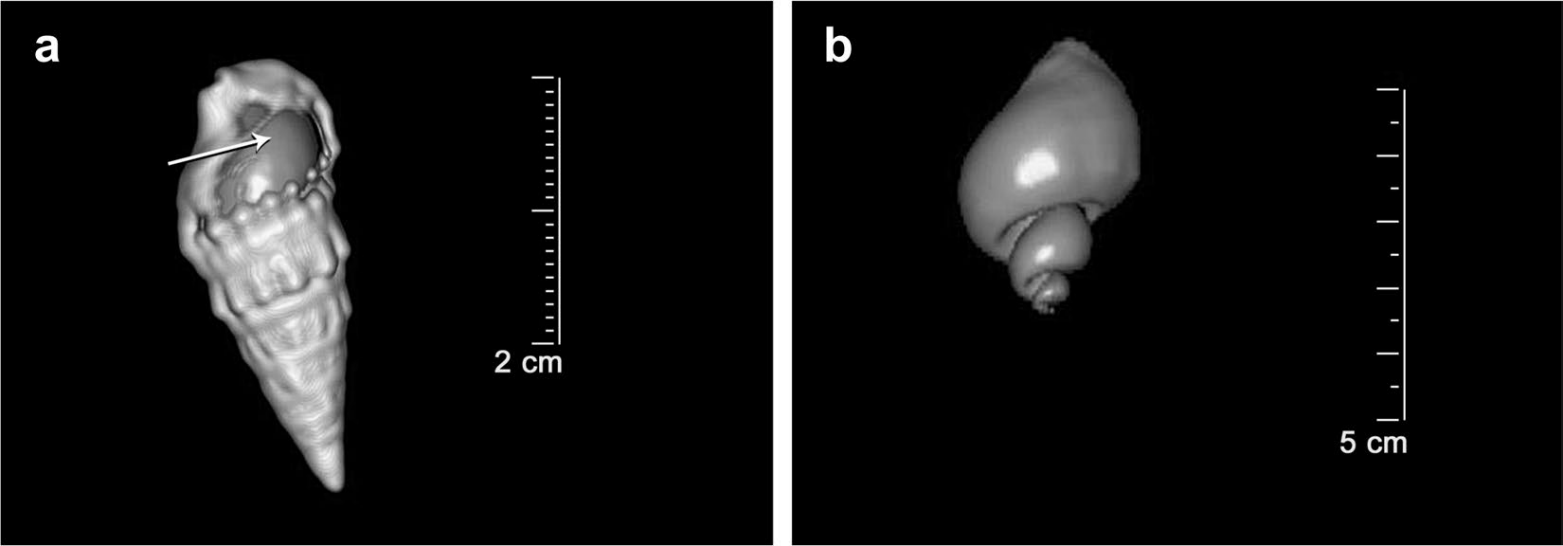
Three-dimensional images reconstructed by CT Viewer software of: (**a**) a *Cerithium atratum* shell showing the volume of air that fills the shell cavity (arrow) and (**b**) the air volume isolated from the shell cavity of *Stramonita haemastoma*.

After the slices were regrouped, the image of each shell was reconstructed three-dimensionally and the internal volume determined from the volume of air present inside the cavity using a pre-set for air on the CT Viewer software (Fig. 5). Window width (WW) and window level (WL), settings used to control the contrast in the grey-scale CT images^52^, were adjusted to fixed values (width = 1000 HU, level = 650 HU; Hounsfield Units).

### Experimental design and hypotheses tested

**Figure 6:**
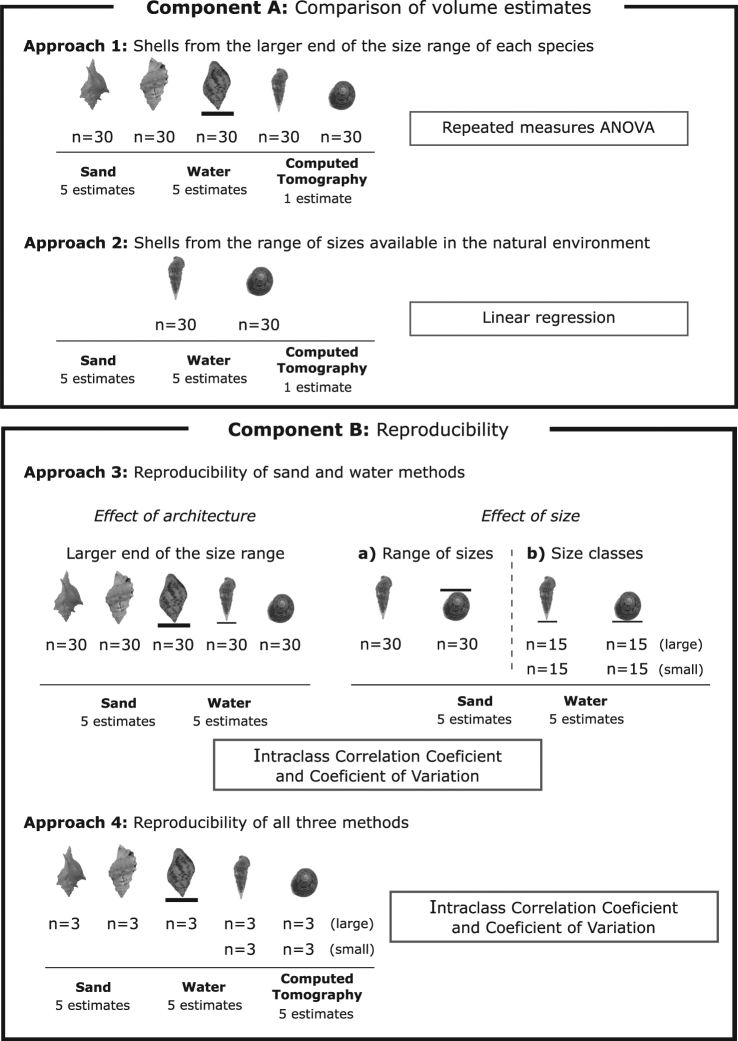
Schematic summary of the experimental design focusing on species used, sample size, repeated measures of volume estimate for each method and statistical analyses used. Note: shell species are not represented in scale. Photographs of *C. senegalensis*, *C. parthenopeum* and *S. haemastoma* were taken by Ragagnin, M.N. and photographs of *C. atratum* and *T. viridula* were reprinted from Dominciano *et al*.^54^ with permission from Elsevier, under license number 243020641674.

The objectives of this study were divided in two components (A and B) each of which comprised two approaches. Component A involved the volume estimates obtained using sand, water and CT methods to determine whether these produced similar volume estimates (separated into approaches 1 and 2). Subsequently, Component B aimed to examine the reproducibility of shell volume estimates obtained using the sand, water and CT methods (separated into approaches 3 and 4). For each approach, shell volume using the sand and water methods was estimated five times by the same team member (MNR) for each specimen to evaluate the reproducibility within, and degree of variation between, methods. Prior to each of the five successive measurements using either sand or water, the specimens were washed and dried in an oven (60 °C for 48 h) and only intact shells (i.e., without damage or perforations) were used. In contrast to the repeated measures obtained using sand and water, CT was performed only once in approaches 1 and 2 because the CT Viewer software provides the volumetric value and calculates the associated standard deviation. However, for approach 4, five volume estimates were made using the CT method to permit a direct comparison of reproducibility with the sand and water methods. Figure 6 shows a schematic summary of the experimental design and analyses used.

### Component A: Comparison of shell volume estimates from three methods

#### Approach 1. Effect of method and shell architecture on volume estimate

The following hypotheses were addressed: (1) there is no variation in the shell volume estimates obtained using sand, water or CT methods; and (2) there is no effect of shell architecture on the shell volume estimates obtained using sand, water or CT methods.

The effect of method and shell architecture on volume estimate was tested using repeated measures Analysis of Variance (ANOVA), which compared the mean values obtained for the three methods and five shell species. For this analysis, the volume of thirty shells from a limited size range at the larger end of the size range of each species was measured to minimize any size effect. Shells with the following average shell length ± SD were used: *C. senegalensis* = 57.9 ± 5.1 mm; *C. parthenopeum* = 52.2 ± 6.3 mm; *S. haemastoma* = 48.0 ± 4.7 mm; *C. atratum* = 28.8 ± 2.2 mm; *T. viridula = *14.0 ± 2.1 mm.

#### Approach 2. Effect of shell architecture and size on volume estimate

The following hypothesis was addressed: (1) there is no effect of shell size on the shell volume estimates obtained using sand, water or CT methods.

The effect of shell size on volume estimates was tested using the two species, which contrasted most in terms of their architecture: *Cerithium atratum* (high-spired) and *Tegula viridula* (low-spired). For both species, thirty shells were selected to represent the range of sizes available in their natural environment (*C. atratum*: average shell length = 21.9 mm, range 8.5 to 34.4 mm; *T. viridula:* average shell length = 10.8 mm, range 3.5 to 15.7 mm). Following log(*x* + 1) transformation of the data, linear regression analysis was used to describe the relationship between volume estimate and shell weight and show the variation in estimates related to shell size among the methods for *C. atratum* and *T. viridula*. For this analysis, weight was chosen in preference to shell length as the feature of length is not comparable between shells of different shape^12^.

### Component B: Examining the degree of reproducibility of shell volume estimates obtained using the three methods

#### Approach 3. Effect of method and shell architecture on reproducibility of volume estimate

The following hypotheses were addressed: (1) Sand and water methods will produce reproducible estimates of shell volume; (2) There is no effect of shell architecture on the reproducibility of shell volume estimates obtained using sand and water methods; and (3) There is no effect of shell size on the reproducibility of shell volume estimates obtained using sand and water methods.

To assess the reproducibility of sand and water methods for shells of different architecture and size, the five replicate volume estimates for the same thirty specimens measured for each species in approaches 1 and 2 were used. Precision for each method was examined to determine if replicate measures gave similar volume estimates within and among methods (i.e., precision is high) and if a single estimate of shell volume (i.e., as is typically used in previous studies) would suffice for shells of different features. This was applied for shells of different architectures (from approach 1) and for shells across a range of sizes for two gastropod species with contrasting shell architecture (from approach 2).

To test the sensitivity to shell size, reproducibility was assessed (a) using the thirty specimens from the full size range of shells for *C. atratum* and *T*. *viridula* from approach 2 and (b) using the same 30 shells but divided in two size classes (n = 15 each) for both species comprising ‘small’ (S) and ‘large’ (L) shells. For *C. atratum*, the average dry weights (g) for S and L shells were 0.25 g (range = 0.04–1.04 g) and 1.63 g (range = 1.06–2.07 g) respectively. For *T. viridula*, the average dry weights (g) for S and L shells were 0.99 g (range = 0.13–2.05 g) and 3.51 g (range = 2.06–5.62 g) respectively.

Reproducibility of shell volume estimates using the sand and water methods was calculated using the Intraclass Correlation Coefficient (ICC) according to Lessells and Boag^53^. This approach uses the between (MS_W_) and among (MS_A_) mean square values from a one-way ANOVA to calculate an ICC value (r) between 0 and 1 (where 1 is equal to perfect reproducibility). In the present study, a one-way ANOVA was used for each species, treating each individual shell as a separate treatment with 5 replicate measures. In addition, the coefficient of variation (CV; (SD *100)/mean) was calculated for each shell specimen in order to provide a measure of the range of variability of shell volume estimates for each shell type.

#### Approach 4. Reproducibility of volume estimates using CT compared to sand and water methods

The following hypothesis was addressed: (1) All three methods (sand, water and CT) will produce reproducible estimates of shell volume.

In Component A, shell volume estimates using CT were only measured once for each shell specimen. Therefore, in order to calculate an ICC value for CT that would enable comparisons among all three methods, replicate shell volume estimates were made using this method. Due to the time and costs involved in making repeated measures for thirty shells of each species, the ICC was calculated for a sub-sample of large shells (n = 3 for each species), selected at random from the 30 shells analyzed in approach 1 and for a sub-sample of small shells (n = 3) from the small sized specimens of both *C. atratum* and *T. viridula* in approach 2. For each of the randomly selected shells (for which 5 repeated estimates had been made using the sand and water methods), five replicate estimates were made using the CT method. Assuming that potential variations in could be caused by the application of clay over the aperture when using the CT method, the clay cover was changed for each of the five estimates. This approach allowed ICC and CV values to be calculated for estimates obtained using CT, which could be compared directly with the ICC and CV values obtained using the sand and water methods for the same specimens.

### Data Availability

All data generated or analyzed during this study are included in this published article (and its Supplementary Information files).

## Electronic supplementary material

Supplementary Information
