# Supplementary material for: Gastropod shell size and architecture influence the applicability of methods used to estimate internal volume
Source: Sci Rep. 2018 Jan 11;8:440. doi: 10.1038/s41598-017-18906-6 (PMC5765162; doi:10.1038/s41598-017-18906-6)
Supplement: Supplementary file 1 — Supplementary Information [file 41598_2017_18906_MOESM1_ESM.pdf]

**Manuscript title: Gastropod shell size and architecture influence the applicability of methods used to estimate internal volume**

**Authors: Marilia Nagata Ragagnin, Daniel Gorman, Ian McCarthy, Bruno Sampaio Sant'Anna, Claudio Campi de Castro, Alexander Turra**

## Approaches 1 & 3

*Chicoreus senegalensis*

| Volume estimates ($cm^3$) - Repeated measures (RM) | | | | | | | |
|---|---|---|---|---|---|---|---|
| Sand Method | | | | | | | |
| specimens | RM 1 | RM 2 | RM 3 | RM 4 | RM 5 | Mean | SD |
| 1 | 3.7017 | 3.7744 | 3.8036 | 3.6826 | 3.5962 | 3.7117 | 0.0816 |
| 2 | 4.6478 | 5.1032 | 5.0666 | 4.4787 | 4.8638 | 4.8320 | 0.2685 |
| 3 | 4.7809 | 4.5483 | 4.7335 | 4.0892 | 4.8369 | 4.5977 | 0.3042 |
| 4 | 6.6678 | 5.8726 | 6.8154 | 5.9275 | 7.0135 | 6.4594 | 0.5255 |
| 5 | 3.5015 | 3.4205 | 3.9568 | 3.6937 | 4.4908 | 3.8126 | 0.4317 |
| 6 | 3.7777 | 4.3626 | 4.1621 | 4.1793 | 4.4673 | 4.1898 | 0.2634 |
| 7 | 6.0074 | 5.4251 | 5.3989 | 5.0376 | 5.9463 | 5.5631 | 0.4081 |
| 8 | 5.9740 | 5.9300 | 6.2638 | 6.3736 | 6.7390 | 6.2561 | 0.3290 |
| 9 | 3.7261 | 4.1561 | 3.9114 | 3.7058 | 4.5478 | 4.0095 | 0.3512 |
| 10 | 5.9587 | 5.8568 | 6.3024 | 5.7987 | 6.4820 | 6.0797 | 0.2978 |
| 11 | 5.3478 | 4.9212 | 4.7465 | 4.9340 | 4.7609 | 4.9421 | 0.2430 |
| 12 | 6.7615 | 6.5675 | 7.0078 | 7.5292 | 7.2608 | 7.0253 | 0.3837 |
| 13 | 5.6710 | 5.2972 | 5.2785 | 5.3222 | 5.6773 | 5.4492 | 0.2059 |
| 14 | 4.1130 | 3.8778 | 4.1461 | 4.1050 | 4.6613 | 4.1806 | 0.2891 |
| 15 | 6.4899 | 5.9907 | 5.6207 | 5.8769 | 7.3213 | 6.2599 | 0.6722 |
| 16 | 3.4758 | 3.4062 | 3.0729 | 3.3712 | 3.9437 | 3.4540 | 0.3141 |
| 17 | 4.3488 | 4.6228 | 4.8583 | 4.4444 | 5.7459 | 4.8041 | 0.5611 |
| 18 | 3.0584 | 2.8503 | 3.2766 | 3.1371 | 3.5982 | 3.1841 | 0.2782 |
| 19 | 3.3206 | 3.1482 | 2.9425 | 3.2679 | 3.8913 | 3.3141 | 0.3539 |
| 20 | 4.7950 | 4.7844 | 4.7309 | 4.9005 | 5.3453 | 4.9112 | 0.2503 |
| 21 | 3.7554 | 3.8025 | 3.6983 | 3.2503 | 4.1445 | 3.7302 | 0.3197 |
| 22 | 4.4561 | 4.3048 | 4.6247 | 4.3660 | 4.6835 | 4.4870 | 0.1631 |
| 23 | 5.2643 | 5.1059 | 5.0609 | 5.4960 | 5.6741 | 5.3202 | 0.2610 |
| 24 | 4.4492 | 4.6835 | 4.6324 | 4.6189 | 5.4638 | 4.7695 | 0.3980 |
| 25 | 7.6921 | 7.6401 | 7.3206 | 7.7355 | 8.0464 | 7.6869 | 0.2588 |
| 26 | 4.3400 | 3.8183 | 4.0970 | 3.9708 | 5.0307 | 4.2513 | 0.4757 |
| 27 | 5.7523 | 5.5529 | 5.4044 | 5.7551 | 6.3623 | 5.7654 | 0.3647 |
| 28 | 3.8960 | 3.8159 | 4.5422 | 4.1139 | 4.8247 | 4.2386 | 0.4322 |
| 29 | 4.0133 | 3.8800 | 3.5979 | 3.5011 | 4.3318 | 3.8648 | 0.3331 |
| 30 | 4.0678 | 4.2714 | 4.0252 | 4.3951 | 5.1943 | 4.3908 | 0.4738 |

| Water Method | | | | | | | |
|---|---|---|---|---|---|---|---|
| specimens | RM 1 | RM 2 | RM 3 | RM 4 | RM 5 | Mean | SD |
| 1 | 3.3181 | 4.2500 | 4.0804 | 4.4182 | 3.9712 | 4.0076 | 0.4211 |

| | | | | | | | |
|---|---|---|---|---|---|---|---|
| 2 | 5.7419 | 5.6684 | 5.4196 | 5.3913 | 5.2390 | 5.4920 | 0.2079 |
| 3 | 5.7546 | 5.5618 | 5.5066 | 5.3741 | 5.5389 | 5.5472 | 0.1368 |
| 4 | 7.5031 | 7.5488 | 6.7663 | 7.6671 | 7.5958 | 7.4162 | 0.3683 |
| 5 | 4.9564 | 4.8282 | 4.8728 | 4.8140 | 4.4988 | 4.7940 | 0.1741 |
| 6 | 4.7629 | 4.7490 | 4.6969 | 4.7893 | 4.4095 | 4.6815 | 0.1557 |
| 7 | 6.7581 | 6.5939 | 6.6633 | 6.7277 | 6.5554 | 6.6597 | 0.0859 |
| 8 | 7.4695 | 7.4546 | 7.4152 | 7.4052 | 7.4103 | 7.4310 | 0.0291 |
| 9 | 4.6626 | 4.2245 | 4.5616 | 4.6756 | 4.3895 | 4.5028 | 0.1931 |
| 10 | 6.8036 | 6.7052 | 6.7578 | 6.8106 | 5.9807 | 6.6116 | 0.3552 |
| 11 | 5.5550 | 5.7923 | 5.5541 | 5.7921 | 4.9066 | 5.5200 | 0.3629 |
| 12 | 8.1447 | 8.1343 | 8.2114 | 7.7122 | 7.9080 | 8.0221 | 0.2077 |
| 13 | 6.2516 | 6.2585 | 6.1440 | 6.3127 | 5.9940 | 6.1922 | 0.1265 |
| 14 | 4.6532 | 4.7012 | 4.7656 | 4.8784 | 4.4605 | 4.6918 | 0.1544 |
| 15 | 7.1465 | 7.2580 | 7.5538 | 7.3596 | 6.6181 | 7.1872 | 0.3517 |
| 16 | 3.9991 | 4.0341 | 3.9972 | 3.8959 | 3.7497 | 3.9352 | 0.1158 |
| 17 | 5.6204 | 5.5144 | 5.5826 | 5.5339 | 5.9090 | 5.6321 | 0.1603 |
| 18 | 3.6422 | 3.6291 | 3.5795 | 3.7442 | 3.6591 | 3.6508 | 0.0600 |
| 19 | 3.4994 | 3.7888 | 3.9596 | 3.5932 | 3.9422 | 3.7566 | 0.2059 |
| 20 | 5.4831 | 5.5802 | 5.8287 | 5.9853 | 5.5739 | 5.6902 | 0.2090 |
| 21 | 4.2257 | 4.1817 | 4.1965 | 4.2166 | 4.3216 | 4.2284 | 0.0548 |
| 22 | 4.9663 | 5.2181 | 5.2195 | 5.3023 | 4.5026 | 5.0418 | 0.3267 |
| 23 | 5.7823 | 5.5350 | 5.8270 | 5.9596 | 5.3658 | 5.6939 | 0.2393 |
| 24 | 5.6271 | 5.5689 | 5.9290 | 5.7580 | 5.4565 | 5.6679 | 0.1820 |
| 25 | 9.2205 | 9.2557 | 9.3807 | 9.4292 | 8.3070 | 9.1186 | 0.4618 |
| 26 | 4.9695 | 5.0867 | 5.0416 | 5.1243 | 5.0828 | 5.0610 | 0.0589 |
| 27 | 6.8202 | 6.6979 | 6.7051 | 6.7451 | 5.6790 | 6.5295 | 0.4779 |
| 28 | 5.0399 | 5.1443 | 5.1088 | 5.1829 | 5.0153 | 5.0982 | 0.0701 |
| 29 | 4.7090 | 4.7489 | 4.6045 | 4.8639 | 4.4894 | 4.6831 | 0.1426 |
| 30 | 5.1884 | 5.3150 | 5.3435 | 5.1899 | 4.7686 | 5.1611 | 0.2305 |

| CT method | | |
|---|---|---|
| specimens | RM 1 | |
| | Mean | SD |
| 1 | 3.1762 | 0.1721 |
| 2 | 4.3290 | 0.2137 |
| 3 | 4.2353 | 0.2078 |
| 4 | 5.8000 | 0.3000 |
| 5 | 3.6395 | 0.1883 |
| 6 | 3.4636 | 0.1763 |
| 7 | 5.0000 | 0.2000 |
| 8 | 5.6000 | 0.3000 |
| 9 | 3.4459 | 0.1802 |
| 10 | 5.2000 | 0.2000 |
| 11 | 4.4714 | 0.2117 |
| 12 | 6.5000 | 0.3000 |
| 13 | 4.6109 | 0.2224 |
| 14 | 3.4802 | 0.1821 |
| 15 | 5.9000 | 0.3000 |

| | | |
|---|---|---|
| 16 | 2.9063 | 0.1449 |
| 17 | 4.1665 | 0.1836 |
| 18 | 2.6490 | 0.1370 |
| 19 | 2.8862 | 0.1415 |
| 20 | 4.3899 | 0.1952 |
| 21 | 3.0830 | 0.1485 |
| 22 | 3.8058 | 0.1771 |
| 23 | 4.4804 | 0.1955 |
| 24 | 4.2770 | 0.1855 |
| 25 | 6.4000 | 0.2000 |
| 26 | 3.6403 | 0.1714 |
| 27 | 5.1000 | 0.2000 |
| 28 | 3.7105 | 0.1728 |
| 29 | 3.4437 | 0.1652 |
| 30 | 3.8157 | 0.1760 |

*Cymatium parthenopeum*

| **Volume estimates (cm³) - Repeated measures (RM)** | | | | | | | |
|---|---|---|---|---|---|---|---|
| Sand Method | | | | | | | |
| specimens | RM 1 | RM 2 | RM 3 | RM 4 | RM 5 | Mean | SD |
| 1 | 6.8352 | 6.0027 | 5.7448 | 6.0639 | 6.8766 | 6.3046 | 0.5175 |
| 2 | 5.1455 | 6.1081 | 5.5389 | 5.7424 | 7.5274 | 6.0125 | 0.9156 |
| 3 | 3.6032 | 3.4486 | 3.3879 | 2.7257 | 4.2565 | 3.4844 | 0.5469 |
| 4 | 5.7938 | 5.5200 | 5.6718 | 5.8281 | 6.4145 | 5.8457 | 0.3402 |
| 5 | 8.8237 | 8.3957 | 8.7108 | 7.7586 | 9.5111 | 8.6400 | 0.6392 |
| 6 | 5.9861 | 5.4734 | 5.6193 | 5.7314 | 5.5960 | 5.6812 | 0.1935 |
| 7 | 3.1098 | 2.9564 | 3.7854 | 3.6407 | 4.0805 | 3.5146 | 0.4703 |
| 8 | 4.3390 | 3.9691 | 4.5594 | 3.9662 | 4.7706 | 4.3208 | 0.3567 |
| 9 | 4.3676 | 4.2471 | 4.6257 | 4.9627 | 5.5900 | 4.7586 | 0.5397 |
| 10 | 11.6409 | 9.8286 | 12.0202 | 10.7451 | 12.5363 | 11.3542 | 1.0749 |
| 11 | 7.3053 | 6.4784 | 6.4775 | 7.8621 | 6.3235 | 6.8894 | 0.6667 |
| 12 | 3.2430 | 3.4001 | 3.1646 | 2.8242 | 4.1318 | 3.3527 | 0.4837 |
| 13 | 8.3765 | 8.1727 | 8.1312 | 8.6998 | 8.2157 | 8.3192 | 0.2322 |
| 14 | 5.0229 | 4.7579 | 4.5010 | 5.3424 | 6.2225 | 5.1693 | 0.6664 |
| 15 | 8.0207 | 7.8801 | 7.6152 | 7.7790 | 9.2860 | 8.1162 | 0.6704 |
| 16 | 4.2661 | 3.5406 | 3.6139 | 3.7946 | 4.0743 | 3.8579 | 0.3073 |
| 17 | 14.3650 | 13.5070 | 15.1922 | 14.5208 | 15.5667 | 14.6303 | 0.7964 |
| 18 | 3.8685 | 3.3027 | 3.5957 | 3.5721 | 4.3025 | 3.7283 | 0.3783 |
| 19 | 3.5977 | 3.4887 | 3.4061 | 3.6552 | 4.2092 | 3.6714 | 0.3157 |
| 20 | 4.6176 | 4.4423 | 4.3426 | 4.5708 | 4.8443 | 4.5635 | 0.1906 |
| 21 | 5.9972 | 5.0960 | 5.5381 | 5.9033 | 6.6939 | 5.8457 | 0.5921 |
| 22 | 4.8771 | 4.4593 | 4.3518 | 4.6769 | 5.7159 | 4.8162 | 0.5421 |
| 23 | 7.3972 | 6.5467 | 6.6873 | 7.2681 | 8.9874 | 7.3773 | 0.9709 |
| 24 | 7.0590 | 7.4567 | 8.0977 | 7.4880 | 7.9005 | 7.6004 | 0.4074 |
| 25 | 5.8617 | 6.1036 | 6.4814 | 6.2008 | 6.9538 | 6.3203 | 0.4180 |
| 26 | 4.1416 | 4.2949 | 3.8096 | 4.5279 | 4.7888 | 4.3126 | 0.3727 |

| | | | | | | | |
|---|---|---|---|---|---|---|---|
| 27 | 3.8294 | 3.8902 | 3.8787 | 3.7741 | 3.9902 | 3.8725 | 0.0802 |
| 28 | 6.3509 | 6.8622 | 6.8346 | 6.4071 | 7.1151 | 6.7140 | 0.3254 |
| 29 | 3.4653 | 3.6274 | 3.0653 | 3.4156 | 3.5627 | 3.4272 | 0.2185 |
| 30 | 4.2111 | 3.7661 | 3.8330 | 4.3542 | 4.0386 | 4.0406 | 0.2479 |

Water Method

| specimens | RM 1 | RM 2 | RM 3 | RM 4 | RM 5 | Mean | SD |
|---|---|---|---|---|---|---|---|
| 1 | 8.0480 | 8.0188 | 8.1776 | 8.2523 | 8.0581 | 8.1110 | 0.0996 |
| 2 | 7.8320 | 8.0924 | 7.9799 | 7.7306 | 8.0244 | 7.9319 | 0.1476 |
| 3 | 4.6570 | 4.4905 | 4.5911 | 4.6679 | 4.5021 | 4.5817 | 0.0834 |
| 4 | 6.8685 | 6.9390 | 6.7808 | 7.0518 | 6.9029 | 6.9086 | 0.0993 |
| 5 | 11.1454 | 11.0369 | 11.2992 | 11.0297 | 11.4208 | 11.1864 | 0.1705 |
| 6 | 6.8533 | 6.8706 | 6.7850 | 6.8428 | 6.6328 | 6.7969 | 0.0972 |
| 7 | 4.6732 | 4.6152 | 4.7568 | 4.7547 | 4.7641 | 4.7128 | 0.0660 |
| 8 | 5.5694 | 5.0910 | 5.5398 | 5.7648 | 4.9522 | 5.3834 | 0.3449 |
| 9 | 6.0498 | 5.5050 | 6.1580 | 6.1152 | 6.2123 | 6.0081 | 0.2874 |
| 10 | 13.4104 | 13.8975 | 13.8729 | 14.0505 | 10.4747 | 13.1412 | 1.5097 |
| 11 | 7.3466 | 8.5104 | 7.6775 | 8.6504 | 8.5580 | 8.1486 | 0.5949 |
| 12 | 4.3815 | 4.4989 | 4.4564 | 4.4042 | 4.2785 | 4.4039 | 0.0837 |
| 13 | 9.2144 | 9.4202 | 9.2920 | 9.4597 | 9.5008 | 9.3774 | 0.1201 |
| 14 | 7.0239 | 6.4081 | 6.9045 | 7.2073 | 6.5420 | 6.8172 | 0.3338 |
| 15 | 9.6006 | 9.9556 | 10.0519 | 9.9682 | 7.7985 | 9.4750 | 0.9531 |
| 16 | 4.7838 | 5.0334 | 5.1104 | 5.0738 | 4.5728 | 4.9148 | 0.2301 |
| 17 | 19.3911 | 18.2223 | 19.7296 | 19.1985 | 14.7913 | 18.2666 | 2.0219 |
| 18 | 4.9676 | 5.0512 | 4.9502 | 5.0718 | 4.8348 | 4.9751 | 0.0942 |
| 19 | 4.7417 | 4.9657 | 4.8207 | 4.9751 | 4.9300 | 4.8866 | 0.1016 |
| 20 | 5.5534 | 5.4917 | 5.5707 | 5.5532 | 5.4530 | 5.5244 | 0.0500 |
| 21 | 8.5897 | 7.2313 | 8.3024 | 8.4711 | 7.1950 | 7.9579 | 0.6876 |
| 22 | 5.8497 | 5.9198 | 6.0064 | 5.8187 | 4.9984 | 5.7186 | 0.4090 |
| 23 | 9.9214 | 9.8974 | 10.1993 | 10.1359 | 10.2889 | 10.0886 | 0.1726 |
| 24 | 9.7134 | 9.7837 | 9.7045 | 9.9607 | 7.8307 | 9.3986 | 0.8825 |
| 25 | 7.7747 | 7.8870 | 7.6731 | 7.4765 | 6.8510 | 7.5325 | 0.4098 |
| 26 | 5.4893 | 5.3374 | 5.4466 | 5.4901 | 5.6126 | 5.4752 | 0.0989 |
| 27 | 4.5004 | 4.5341 | 4.6552 | 4.7495 | 4.6484 | 4.6175 | 0.1006 |
| 28 | 7.8152 | 7.9111 | 8.0459 | 7.7990 | 7.6754 | 7.8493 | 0.1382 |
| 29 | 3.8803 | 3.9108 | 3.9233 | 4.0265 | 3.7528 | 3.8987 | 0.0984 |
| 30 | 5.2037 | 5.0482 | 5.5180 | 5.0544 | 5.4923 | 5.2633 | 0.2295 |

CT method

| specimens | RM 1 | |
|---|---|---|
| | Mean | SD |
| 1 | 6.5000 | 0.3000 |
| 2 | 6.0000 | 0.3000 |
| 3 | 3.4116 | 0.2075 |
| 4 | 5.4000 | 0.3000 |
| 5 | 8.9000 | 0.4000 |
| 6 | 5.6000 | 0.3000 |

| | | |
|---|---|---|
| 7 | 3.3763 | 0.2068 |
| 8 | 4.2704 | 0.2361 |
| 9 | 4.7267 | 0.2433 |
| 10 | 11.3000 | 0.4000 |
| 11 | 6.6000 | 0.3000 |
| 12 | 3.1225 | 0.1975 |
| 13 | 7.6000 | 0.3000 |
| 14 | 5.4000 | 0.3000 |
| 15 | 7.9000 | 0.4000 |
| 16 | 3.8658 | 0.1903 |
| 17 | 15.7000 | 0.5000 |
| 18 | 3.6322 | 0.1898 |
| 19 | 3.8086 | 0.1904 |
| 20 | 4.4298 | 0.2148 |
| 21 | 6.5000 | 0.3000 |
| 22 | 4.6720 | 0.2165 |
| 23 | 8.0000 | 0.3000 |
| 24 | 8.0000 | 0.3000 |
| 25 | 6.3000 | 0.3000 |
| 26 | 4.2512 | 0.2019 |
| 27 | 3.5952 | 0.1840 |
| 28 | 6.2000 | 0.3000 |
| 29 | 2.9033 | 0.1562 |
| 30 | 4.0994 | 0.2171 |

*Stramonita haemastoma*

| Volume estimates (cm³) - Repeated measures (RM) | | | | | | | |
|---|---|---|---|---|---|---|---|
| Sand Method | | | | | | | |
| specimens | RM 1 | RM 2 | RM 3 | RM 4 | RM 5 | Mean | SD |
| 1 | 9.4178 | 8.9581 | 9.0429 | 8.8309 | 8.7331 | 8.9966 | 0.2636 |
| 2 | 6.1098 | 6.3568 | 6.3707 | 6.4965 | 6.1453 | 6.2958 | 0.1634 |
| 3 | 4.1181 | 4.6123 | 4.2114 | 4.2779 | 4.3008 | 4.3041 | 0.1863 |
| 4 | 4.9276 | 5.0734 | 4.6068 | 5.1309 | 5.1496 | 4.9777 | 0.2249 |
| 5 | 6.7147 | 6.5144 | 6.9501 | 6.2062 | 6.5207 | 6.5812 | 0.2751 |
| 6 | 4.9368 | 5.1041 | 5.1280 | 5.0880 | 6.0133 | 5.2540 | 0.4310 |
| 7 | 3.1548 | 3.0573 | 2.8734 | 3.0447 | 3.4925 | 3.1245 | 0.2293 |
| 8 | 7.4099 | 7.8654 | 7.0586 | 7.6356 | 8.2587 | 7.6456 | 0.4540 |
| 9 | 9.1426 | 8.9504 | 9.2395 | 10.2697 | 9.6547 | 9.4514 | 0.5249 |
| 10 | 5.9545 | 5.9107 | 6.6570 | 5.5053 | 6.5146 | 6.1084 | 0.4723 |
| 11 | 3.5263 | 3.7270 | 3.2934 | 4.0100 | 4.3549 | 3.7823 | 0.4145 |
| 12 | 6.6679 | 6.8248 | 6.5783 | 6.0808 | 6.4759 | 6.5255 | 0.2797 |
| 13 | 6.5805 | 6.5614 | 5.9496 | 6.9948 | 6.4545 | 6.5082 | 0.3741 |
| 14 | 8.9215 | 8.3515 | 8.1661 | 8.1034 | 8.2197 | 8.3525 | 0.3310 |
| 15 | 3.9633 | 4.0028 | 3.7325 | 3.6830 | 4.0795 | 3.8922 | 0.1744 |
| 16 | 4.1592 | 4.2726 | 4.5414 | 4.0975 | 3.9524 | 4.2046 | 0.2209 |
| 17 | 3.9717 | 3.9887 | 4.0563 | 3.8289 | 4.1269 | 3.9945 | 0.1110 |

| | | | | | | | |
|---|---|---|---|---|---|---|---|
| 18 | 3.3283 | 3.3963 | 3.4670 | 3.3944 | 3.6598 | 3.4492 | 0.1276 |
| 19 | 3.6512 | 3.4739 | 3.3651 | 3.6040 | 3.6020 | 3.5392 | 0.1175 |
| 20 | 5.5754 | 5.1155 | 5.7271 | 5.5269 | 5.5756 | 5.5041 | 0.2299 |
| 21 | 6.4762 | 6.0924 | 6.6462 | 5.7737 | 6.3336 | 6.2644 | 0.3413 |
| 22 | 7.5708 | 7.7786 | 7.2734 | 7.4304 | 6.8653 | 7.3837 | 0.3443 |
| 23 | 8.3558 | 8.0584 | 7.8136 | 8.2039 | 8.3903 | 8.1644 | 0.2364 |
| 24 | 5.0096 | 4.6103 | 5.3830 | 4.8334 | 5.6643 | 5.1001 | 0.4235 |
| 25 | 3.3299 | 3.2360 | 3.3247 | 3.2998 | 3.8485 | 3.4078 | 0.2492 |
| 26 | 10.1719 | 9.9199 | 9.8053 | 9.8258 | 10.4126 | 10.0271 | 0.2602 |
| 27 | 3.5935 | 3.3796 | 2.9618 | 3.2151 | 3.3545 | 3.3009 | 0.2329 |
| 28 | 6.1481 | 6.4026 | 6.3194 | 6.1765 | 6.2597 | 6.2613 | 0.1041 |
| 29 | 8.4713 | 8.8092 | 8.1769 | 8.5023 | 8.1896 | 8.4299 | 0.2610 |
| 30 | 6.5258 | 6.5977 | 6.4616 | 6.7656 | 6.5599 | 6.5821 | 0.1141 |

| Water Method | | | | | | | |
|---|---|---|---|---|---|---|---|
| specimens | RM 1 | RM 2 | RM 3 | RM 4 | RM 5 | Mean | SD |
| 1 | 9.8212 | 9.7815 | 9.8076 | 10.0047 | 9.0496 | 9.6929 | 0.3703 |
| 2 | 6.8933 | 6.7167 | 6.8894 | 6.7947 | 6.6326 | 6.7853 | 0.1125 |
| 3 | 4.9335 | 4.7907 | 5.0457 | 4.9980 | 5.1526 | 4.9841 | 0.1345 |
| 4 | 5.8275 | 5.5490 | 5.9373 | 5.5380 | 5.7968 | 5.7297 | 0.1779 |
| 5 | 6.9652 | 6.9703 | 6.9041 | 6.8066 | 7.0628 | 6.9418 | 0.0945 |
| 6 | 5.7472 | 5.9335 | 5.8540 | 5.8360 | 5.9552 | 5.8652 | 0.0832 |
| 7 | 3.6620 | 3.6579 | 3.6598 | 3.6351 | 3.5615 | 3.6353 | 0.0426 |
| 8 | 8.0952 | 8.0765 | 8.1187 | 7.8555 | 8.2395 | 8.0771 | 0.1392 |
| 9 | 9.3115 | 9.7411 | 10.0819 | 9.8105 | 10.1827 | 9.8255 | 0.3409 |
| 10 | 6.8584 | 7.0564 | 7.0167 | 6.8195 | 6.7616 | 6.9025 | 0.1279 |
| 11 | 3.4997 | 4.3505 | 4.3436 | 4.3739 | 4.3114 | 4.1758 | 0.3786 |
| 12 | 6.8675 | 7.3182 | 7.1662 | 7.0742 | 7.3589 | 7.1570 | 0.1984 |
| 13 | 7.0584 | 7.0929 | 7.2354 | 6.9218 | 7.1228 | 7.0863 | 0.1134 |
| 14 | 8.5881 | 8.5178 | 9.0260 | 8.7356 | 9.0018 | 8.7739 | 0.2330 |
| 15 | 3.8912 | 4.3377 | 4.2637 | 4.2033 | 4.2495 | 4.1891 | 0.1734 |
| 16 | 4.6801 | 4.5175 | 3.8344 | 4.3924 | 4.6000 | 4.4049 | 0.3362 |
| 17 | 4.3792 | 4.1108 | 4.2927 | 4.1409 | 4.3818 | 4.2611 | 0.1290 |
| 18 | 3.1702 | 3.7274 | 3.8500 | 3.7612 | 3.8807 | 3.6779 | 0.2906 |
| 19 | 4.0251 | 3.9093 | 3.8452 | 3.6850 | 3.9138 | 3.8757 | 0.1247 |
| 20 | 5.6801 | 6.0210 | 6.0507 | 5.9795 | 6.1062 | 5.9675 | 0.1672 |
| 21 | 6.7614 | 6.6060 | 6.9090 | 6.6512 | 6.8562 | 6.7568 | 0.1294 |
| 22 | 7.7814 | 7.7453 | 7.9667 | 7.9563 | 7.8791 | 7.8658 | 0.1002 |
| 23 | 8.9721 | 8.7011 | 8.8402 | 8.8625 | 8.5300 | 8.7812 | 0.1703 |
| 24 | 6.0399 | 6.2382 | 6.0950 | 5.8692 | 6.0483 | 6.0581 | 0.1322 |
| 25 | 3.7217 | 3.7566 | 3.8708 | 3.8753 | 3.8663 | 3.8181 | 0.0732 |
| 26 | 10.2193 | 10.6797 | 10.2348 | 10.1937 | 9.4506 | 10.1556 | 0.4426 |
| 27 | 3.1198 | 3.4964 | 3.4998 | 3.5567 | 3.7280 | 3.4801 | 0.2224 |
| 28 | 6.9333 | 6.6268 | 6.6190 | 6.7777 | 6.4151 | 6.6744 | 0.1938 |
| 29 | 8.4775 | 8.5280 | 9.1600 | 8.7166 | 9.4353 | 8.8635 | 0.4177 |
| 30 | 6.9552 | 6.9019 | 7.1881 | 7.1080 | 9.6426 | 7.5592 | 1.1703 |

| CT method | | |
|---|---|---|
| specimens | RM 1 | |
| | Mean | SD |
| 1 | 7.8000 | 0.3000 |
| 2 | 5.7000 | 0.3000 |
| 3 | 3.8532 | 0.2067 |
| 4 | 4.4510 | 0.2354 |
| 5 | 5.5000 | 0.3000 |
| 6 | 4.4146 | 0.2258 |
| 7 | 2.6186 | 0.1611 |
| 8 | 6.5000 | 0.3000 |
| 9 | 8.2000 | 0.3000 |
| 10 | 5.5000 | 0.3000 |
| 11 | 3.2620 | 0.1833 |
| 12 | 5.6000 | 0.3000 |
| 13 | 5.6000 | 0.3000 |
| 14 | 7.1000 | 0.3000 |
| 15 | 3.2498 | 0.1827 |
| 16 | 3.5285 | 0.1751 |
| 17 | 3.3926 | 0.1709 |
| 18 | 2.8544 | 0.1543 |
| 19 | 3.0026 | 0.1501 |
| 20 | 4.8410 | 0.2195 |
| 21 | 5.3000 | 0.2000 |
| 22 | 6.3000 | 0.3000 |
| 23 | 7.0000 | 0.3000 |
| 24 | 4.6484 | 0.2166 |
| 25 | 2.9635 | 0.1582 |
| 26 | 8.5000 | 0.3000 |
| 27 | 2.6787 | 0.1453 |
| 28 | 5.5000 | 0.3000 |
| 29 | 7.4000 | 0.3000 |
| 30 | 5.7000 | 0.3000 |

*Cerithium atratum*

| **Volume estimates (cm³) - Repeated measures (RM)** | | | | | | | |
|---|---|---|---|---|---|---|---|
| Sand Method | | | | | | | |
| specimens | RM 1 | RM 2 | RM 3 | RM 4 | RM 5 | Mean | SD |
| 1 | 0.7856 | 0.7394 | 0.8523 | 0.9314 | 0.8181 | 0.8254 | 0.0724 |
| 2 | 0.7401 | 0.8355 | 0.6480 | 0.6821 | 0.6809 | 0.7173 | 0.0739 |
| 3 | 0.3212 | 0.3170 | 0.4358 | 0.4408 | 0.3347 | 0.3699 | 0.0628 |
| 4 | 0.6781 | 0.5364 | 0.5951 | 0.6030 | 0.5379 | 0.5901 | 0.0582 |
| 5 | 0.8810 | 0.7233 | 0.8469 | 0.7539 | 0.7606 | 0.7932 | 0.0673 |
| 6 | 0.7280 | 0.7647 | 0.7178 | 0.6973 | 0.7043 | 0.7224 | 0.0265 |
| 7 | 0.5362 | 0.5050 | 0.5162 | 0.5272 | 0.5348 | 0.5239 | 0.0132 |

| | | | | | | | |
|---|---|---|---|---|---|---|---|
| 8 | 0.4492 | 0.4113 | 0.3349 | 0.4464 | 0.4670 | 0.4217 | 0.0526 |
| 9 | 0.4363 | 0.4082 | 0.3990 | 0.4183 | 0.3424 | 0.4008 | 0.0355 |
| 10 | 0.3878 | 0.4090 | 0.3470 | 0.3007 | 0.2598 | 0.3409 | 0.0614 |
| 11 | 0.5790 | 0.5376 | 0.3938 | 0.5480 | 0.3704 | 0.4858 | 0.0962 |
| 12 | 0.4487 | 0.4099 | 0.4271 | 0.4227 | 0.3804 | 0.4178 | 0.0251 |
| 13 | 0.6111 | 0.6170 | 0.5790 | 0.5596 | 0.4497 | 0.5633 | 0.0677 |
| 14 | 0.7147 | 0.6884 | 0.6245 | 0.5355 | 0.5699 | 0.6266 | 0.0760 |
| 15 | 0.3702 | 0.3506 | 0.3539 | 0.3667 | 0.2616 | 0.3406 | 0.0449 |
| 16 | 0.6988 | 0.6640 | 0.6351 | 0.6352 | 0.6124 | 0.6491 | 0.0333 |
| 17 | 0.2231 | 0.2257 | 0.1481 | 0.2318 | 0.1662 | 0.1990 | 0.0388 |
| 18 | 0.7899 | 0.7797 | 1.1874 | 0.6670 | 0.5592 | 0.7966 | 0.2378 |
| 19 | 0.5734 | 0.6171 | 0.5745 | 0.5765 | 0.3674 | 0.5418 | 0.0992 |
| 20 | 0.5773 | 0.6031 | 0.5869 | 0.6206 | 0.3548 | 0.5485 | 0.1096 |
| 21 | 0.9613 | 0.8666 | 0.8123 | 0.9015 | 0.6986 | 0.8481 | 0.0995 |
| 22 | 0.7851 | 0.7750 | 0.7586 | 0.8168 | 0.4322 | 0.7135 | 0.1587 |
| 23 | 0.6185 | 0.6260 | 0.5477 | 0.6628 | 0.3884 | 0.5687 | 0.1091 |
| 24 | 0.5849 | 0.5811 | 0.5463 | 0.5484 | 0.3502 | 0.5222 | 0.0978 |
| 25 | 0.7286 | 0.7135 | 0.6734 | 0.6403 | 0.4883 | 0.6488 | 0.0962 |
| 26 | 0.6129 | 0.6264 | 0.6330 | 0.6408 | 0.4361 | 0.5899 | 0.0866 |
| 27 | 0.5772 | 0.5851 | 0.5656 | 0.5472 | 0.4017 | 0.5354 | 0.0761 |
| 28 | 0.1349 | 0.4247 | 0.3947 | 0.4162 | 0.2768 | 0.3295 | 0.1240 |
| 29 | 0.6113 | 0.6020 | 0.5848 | 0.5599 | 0.4870 | 0.5690 | 0.0498 |
| 30 | 0.7906 | 0.7936 | 0.7289 | 0.7756 | 0.7535 | 0.7684 | 0.0272 |

| Water Method | | | | | | | |
|---|---|---|---|---|---|---|---|
| specimens | RM 1 | RM 2 | RM 3 | RM 4 | RM 5 | Mean | SD |
| 1 | 0.7709 | 1.0312 | 0.8658 | 0.8956 | 0.9079 | 0.8943 | 0.0935 |
| 2 | 0.4650 | 0.8074 | 0.7927 | 0.8369 | 0.9067 | 0.7617 | 0.1716 |
| 3 | 0.5536 | 0.4769 | 0.4207 | 0.4747 | 0.4626 | 0.4777 | 0.0481 |
| 4 | 0.6112 | 0.7168 | 0.7133 | 0.6973 | 0.6824 | 0.6842 | 0.0431 |
| 5 | 0.8170 | 0.7602 | 0.7981 | 0.9895 | 1.1685 | 0.9067 | 0.1708 |
| 6 | 0.7320 | 0.7480 | 0.6652 | 0.8382 | 0.5462 | 0.7059 | 0.1085 |
| 7 | 0.4089 | 0.4868 | 0.4144 | 0.5354 | 0.3985 | 0.4488 | 0.0597 |
| 8 | 0.5078 | 0.4869 | 0.5223 | 0.5420 | 0.5148 | 0.5148 | 0.0201 |
| 9 | 0.2990 | 0.3949 | 0.4310 | 0.2731 | 0.3551 | 0.3506 | 0.0654 |
| 10 | 0.4030 | 0.3796 | 0.3935 | 0.3923 | 0.3341 | 0.3805 | 0.0272 |
| 11 | 0.6870 | 0.6579 | 0.6040 | 0.5644 | 0.5440 | 0.6115 | 0.0606 |
| 12 | 0.2781 | 0.3291 | 0.4719 | 0.4996 | 0.4881 | 0.4134 | 0.1023 |
| 13 | 0.4703 | 0.5439 | 0.6551 | 0.5969 | 0.6206 | 0.5774 | 0.0722 |
| 14 | 0.7077 | 0.5907 | 0.7918 | 0.6755 | 0.8273 | 0.7186 | 0.0942 |
| 15 | 0.2687 | 0.2661 | 0.3267 | 0.2785 | 0.4230 | 0.3126 | 0.0664 |
| 16 | 0.5719 | 0.7729 | 0.7147 | 0.5839 | 0.8260 | 0.6939 | 0.1130 |
| 17 | 0.1683 | 0.2488 | 0.2597 | 0.2693 | 0.2445 | 0.2381 | 0.0402 |
| 18 | 0.7001 | 0.8260 | 0.6703 | 0.5908 | 0.7688 | 0.7112 | 0.0905 |
| 19 | 0.4525 | 0.5755 | 0.6031 | 0.4641 | 0.2834 | 0.4757 | 0.1263 |
| 20 | 0.6519 | 0.6144 | 0.6190 | 0.7020 | 0.3627 | 0.5900 | 0.1318 |
| 21 | 0.9456 | 0.9458 | 1.0601 | 1.0614 | 0.9409 | 0.9908 | 0.0639 |

| | | | | | | | |
|---|---|---|---|---|---|---|---|
| 22 | 0.8185 | 0.9748 | 0.9020 | 0.7917 | 0.6796 | 0.8333 | 0.1122 |
| 23 | 0.6138 | 0.6592 | 0.6219 | 0.7746 | 0.6599 | 0.6659 | 0.0643 |
| 24 | 0.4663 | 0.6346 | 0.4318 | 0.5458 | 0.6238 | 0.5405 | 0.0910 |
| 25 | 0.6690 | 0.6943 | 0.6536 | 0.8620 | 0.5829 | 0.6924 | 0.1035 |
| 26 | 0.5660 | 0.6057 | 0.6630 | 0.5381 | 0.5670 | 0.5880 | 0.0484 |
| 27 | 0.6304 | 0.5684 | 0.6616 | 0.7044 | 0.3004 | 0.5730 | 0.1603 |
| 28 | 0.4203 | 0.4839 | 0.4272 | 0.5034 | 0.3677 | 0.4405 | 0.0541 |
| 29 | 0.4926 | 0.5492 | 0.6920 | 0.6810 | 0.4841 | 0.5798 | 0.1007 |
| 30 | 0.8368 | 0.8248 | 0.6921 | 0.5823 | 0.3648 | 0.6602 | 0.1953 |

| CT method | | |
|---|---|---|
| specimens | RM 1 | |
| | Mean | SD |
| 1 | 0.6291 | 0.0330 |
| 2 | 0.4799 | 0.0301 |
| 3 | 0.2641 | 0.0214 |
| 4 | 0.4019 | 0.0290 |
| 5 | 0.5044 | 0.0335 |
| 6 | 0.4868 | 0.0612 |
| 7 | 0.2979 | 0.0432 |
| 8 | 0.2503 | 0.0312 |
| 9 | 0.2177 | 0.0189 |
| 10 | 0.1555 | 0.0142 |
| 11 | 0.2352 | 0.0190 |
| 12 | 0.2261 | 0.0191 |
| 13 | 0.2577 | 0.0209 |
| 14 | 0.3910 | 0.0284 |
| 15 | 0.1830 | 0.0159 |
| 16 | 0.4107 | 0.0294 |
| 17 | 0.0978 | 0.0093 |
| 18 | 0.5592 | 0.0660 |
| 19 | 0.3674 | 0.0485 |
| 20 | 0.3548 | 0.0482 |
| 21 | 0.6986 | 0.0668 |
| 22 | 0.4322 | 0.0546 |
| 23 | 0.3884 | 0.0513 |
| 24 | 0.3502 | 0.0483 |
| 25 | 0.4883 | 0.0546 |
| 26 | 0.4361 | 0.0550 |
| 27 | 0.4017 | 0.0521 |
| 28 | 0.2768 | 0.0396 |
| 29 | 0.3512 | 0.0470 |
| 30 | 0.5109 | 0.0610 |

*Tegula viridula*

| Volume estimates (cm³) - Repeated measures (RM) | | | | | | | |
|---|---|---|---|---|---|---|---|
| Sand Method | | | | | | | |
| specimens | RM 1 | RM 2 | RM 3 | RM 4 | RM 5 | Mean | SD |
| 1 | 0.8049 | 0.7940 | 0.8583 | 0.7704 | 0.7718 | 0.7999 | 0.0358 |
| 2 | 1.3520 | 1.2822 | 1.1114 | 1.2683 | 0.9695 | 1.1967 | 0.1545 |
| 3 | 1.0473 | 1.2323 | 0.9714 | 1.0092 | 0.9177 | 1.0356 | 0.1199 |
| 4 | 0.5922 | 0.5378 | 0.5469 | 0.5673 | 0.4680 | 0.5424 | 0.0466 |
| 5 | 1.1270 | 1.1354 | 1.0240 | 1.0323 | 0.8007 | 1.0239 | 0.1350 |
| 6 | 1.4289 | 1.4879 | 1.4707 | 1.4362 | 1.1961 | 1.4040 | 0.1187 |
| 7 | 0.6301 | 0.6430 | 0.6199 | 0.5854 | 0.5132 | 0.5983 | 0.0522 |
| 8 | 0.7032 | 0.6955 | 0.6564 | 0.6081 | 0.5450 | 0.6416 | 0.0659 |
| 9 | 0.5248 | 0.2275 | 0.5816 | 0.5680 | 0.5165 | 0.4837 | 0.1458 |
| 10 | 1.2917 | 1.4303 | 1.0803 | 1.1376 | 1.0309 | 1.1942 | 0.1644 |
| 11 | 1.3508 | 1.3790 | 1.1417 | 1.3154 | 1.1867 | 1.2747 | 0.1046 |
| 12 | 0.6573 | 0.7117 | 0.6423 | 0.7007 | 0.6576 | 0.6739 | 0.0304 |
| 13 | 2.2940 | 2.1392 | 2.1269 | 2.1026 | 1.7968 | 2.0919 | 0.1813 |
| 14 | 1.3573 | 1.3177 | 1.2651 | 1.3797 | 1.1325 | 1.2905 | 0.0984 |
| 15 | 1.4469 | 1.4591 | 1.2474 | 1.4587 | 1.0404 | 1.3305 | 0.1855 |
| 16 | 1.2109 | 1.2143 | 1.1906 | 1.1909 | 0.8938 | 1.1401 | 0.1381 |
| 17 | 1.1863 | 1.2560 | 1.3011 | 1.2635 | 0.8470 | 1.1708 | 0.1857 |
| 18 | 1.4196 | 1.7185 | 1.5462 | 1.4615 | 1.2400 | 1.4772 | 0.1752 |
| 19 | 1.3505 | 1.4485 | 1.6241 | 1.4791 | 1.2692 | 1.4343 | 0.1346 |
| 20 | 1.2586 | 1.1735 | 1.2916 | 1.2663 | 1.1510 | 1.2282 | 0.0619 |
| 21 | 1.2033 | 1.0184 | 1.1135 | 1.0039 | 0.9672 | 1.0613 | 0.0960 |
| 22 | 1.1150 | 1.2440 | 1.2600 | 1.3143 | 1.1642 | 1.2195 | 0.0794 |
| 23 | 1.2642 | 1.2971 | 1.2801 | 1.2172 | 1.2452 | 1.2608 | 0.0310 |
| 24 | 0.8009 | 0.7399 | 0.7740 | 0.7630 | 0.7437 | 0.7643 | 0.0248 |
| 25 | 0.6314 | 0.5964 | 0.6513 | 0.6254 | 0.4972 | 0.6004 | 0.0609 |
| 26 | 0.5851 | 0.6228 | 0.6114 | 0.5417 | 0.5147 | 0.5751 | 0.0460 |
| 27 | 0.6250 | 0.5091 | 0.6120 | 0.6145 | 0.4385 | 0.5598 | 0.0825 |
| 28 | 0.6381 | 0.6298 | 0.6918 | 0.6644 | 0.6328 | 0.6514 | 0.0264 |
| 29 | 0.5190 | 0.5249 | 0.5270 | 0.5564 | 0.4809 | 0.5216 | 0.0270 |
| 30 | 0.6028 | 0.5772 | 0.5461 | 0.5375 | 0.4920 | 0.5511 | 0.0420 |

| Water Method | | | | | | | |
|---|---|---|---|---|---|---|---|
| specimens | RM 1 | RM 2 | RM 3 | RM 4 | RM 5 | Mean | SD |
| 1 | 1.0099 | 0.9511 | 0.9616 | 0.9609 | 0.7031 | 0.9173 | 0.1219 |
| 2 | 1.1916 | 1.2596 | 1.2267 | 1.4198 | 1.0410 | 1.2277 | 0.1361 |
| 3 | 1.0095 | 1.2434 | 1.0080 | 1.1330 | 0.9488 | 1.0685 | 0.1186 |
| 4 | 0.6239 | 0.6294 | 0.4712 | 0.5836 | 0.6105 | 0.5837 | 0.0653 |
| 5 | 1.0629 | 1.0024 | 0.9549 | 1.2091 | 0.8116 | 1.0082 | 0.1457 |
| 6 | 1.4568 | 1.3882 | 1.5252 | 1.5156 | 1.3901 | 1.4552 | 0.0657 |
| 7 | 0.6039 | 0.5566 | 0.6571 | 0.7463 | 0.7279 | 0.6584 | 0.0805 |

| | | | | | | | |
|---|---|---|---|---|---|---|---|
| 8 | 0.7288 | 0.7463 | 0.7297 | 0.7936 | 0.6178 | 0.7232 | 0.0646 |
| 9 | 0.6393 | 0.6630 | 0.6386 | 0.7079 | 0.6512 | 0.6600 | 0.0286 |
| 10 | 1.3362 | 1.3031 | 1.2536 | 1.3344 | 1.4161 | 1.3287 | 0.0592 |
| 11 | 1.1494 | 1.1718 | 1.2953 | 1.3973 | 1.5104 | 1.3048 | 0.1523 |
| 12 | 0.6884 | 0.8104 | 0.7465 | 0.7449 | 0.8584 | 0.7697 | 0.0657 |
| 13 | 2.1616 | 2.3254 | 2.1085 | 2.1984 | 2.0677 | 2.1723 | 0.0991 |
| 14 | 1.2443 | 1.3321 | 1.3252 | 1.3297 | 1.1625 | 1.2788 | 0.0747 |
| 15 | 1.5095 | 1.3547 | 1.4475 | 1.4970 | 1.3945 | 1.4406 | 0.0661 |
| 16 | 1.2274 | 1.2222 | 1.1790 | 1.0981 | 1.2225 | 1.1898 | 0.0549 |
| 17 | 1.3386 | 1.0378 | 1.1884 | 1.2645 | 0.7489 | 1.1156 | 0.2333 |
| 18 | 1.7446 | 1.6438 | 1.3705 | 1.6637 | 1.3421 | 1.5529 | 0.1837 |
| 19 | 1.6227 | 1.4634 | 1.4762 | 1.5592 | 1.4483 | 1.5140 | 0.0744 |
| 20 | 1.2235 | 1.0391 | 1.1865 | 1.2162 | 1.1346 | 1.1600 | 0.0761 |
| 21 | 1.2481 | 1.2554 | 1.0275 | 1.2315 | 1.2155 | 1.1956 | 0.0952 |
| 22 | 1.4010 | 1.2094 | 1.2889 | 1.3694 | 1.1658 | 1.2869 | 0.1006 |
| 23 | 1.1709 | 1.1187 | 1.1049 | 1.2050 | 1.2171 | 1.1633 | 0.0502 |
| 24 | 0.8253 | 0.7427 | 0.7508 | 0.8423 | 0.7264 | 0.7775 | 0.0525 |
| 25 | 0.6259 | 0.6357 | 0.5612 | 0.5037 | 0.5439 | 0.5741 | 0.0559 |
| 26 | 0.4626 | 0.6009 | 0.5826 | 0.7122 | 0.5235 | 0.5764 | 0.0933 |
| 27 | 0.6268 | 0.5299 | 0.5701 | 0.5531 | 0.4802 | 0.5520 | 0.0538 |
| 28 | 0.7642 | 0.5766 | 0.6503 | 0.7069 | 0.5950 | 0.6586 | 0.0780 |
| 29 | 0.5990 | 0.5495 | 0.4616 | 0.5429 | 0.6235 | 0.5553 | 0.0623 |
| 30 | 0.6206 | 0.5202 | 0.5295 | 0.5333 | 0.5817 | 0.5571 | 0.0428 |

| CT method | | |
|---|---|---|
| specimens | RM 1 | |
| | Mean | SD |
| 1 | 0.6045 | 0.0253 |
| 2 | 0.9591 | 0.0339 |
| 3 | 0.8315 | 0.0320 |
| 4 | 0.3632 | 0.0169 |
| 5 | 0.7831 | 0.0307 |
| 6 | 1.1721 | 0.0416 |
| 7 | 0.4405 | 0.0199 |
| 8 | 0.4773 | 0.0212 |
| 9 | 0.3997 | 0.0180 |
| 10 | 0.9702 | 0.0348 |
| 11 | 0.8935 | 0.0341 |
| 12 | 0.4280 | 0.0211 |
| 13 | 1.6445 | 0.1210 |
| 14 | 1.0585 | 0.0873 |
| 15 | 1.0730 | 0.0869 |
| 16 | 0.8449 | 0.0740 |
| 17 | 0.8929 | 0.0771 |
| 18 | 1.1998 | 0.0962 |
| 19 | 1.1783 | 0.0942 |
| 20 | 0.9264 | 0.0795 |
| 21 | 0.8498 | 0.0747 |

| | | |
|---|---|---|
| 22 | 0.9275 | 0.0776 |
| 23 | 0.9026 | 0.0779 |
| 24 | 0.5478 | 0.0548 |
| 25 | 0.4007 | 0.0423 |
| 26 | 0.3622 | 0.0177 |
| 27 | 0.3614 | 0.0391 |
| 28 | 0.4459 | 0.0456 |
| 29 | 0.2758 | 0.0144 |
| 30 | 0.3860 | 0.0408 |

## Approaches 2 & 3

*Cerithium atratum*

| | | Volume estimates ($cm^3$) - Repeated measures (RM) | | | | | | |
|---|---|---|---|---|---|---|---|---|
| | | Sand Method | | | | | | |
| specimens | dry weight (g) | RM 1 | RM 2 | RM 3 | RM 4 | RM 5 | Mean | SD |
| 1 | 0.0554 | 0.0160 | 0.0216 | 0.0135 | 0.0243 | 0.0149 | 0.0181 | 0.0047 |
| 2 | 0.0377 | 0.0159 | 0.0202 | 0.0186 | 0.0218 | 0.0030 | 0.0159 | 0.0075 |
| 3 | 0.2311 | 0.0992 | 0.1078 | 0.0980 | 0.0850 | 0.0258 | 0.0832 | 0.0331 |
| 4 | 0.0827 | 0.0285 | 0.0513 | 0.0439 | 0.0404 | 0.0317 | 0.0392 | 0.0092 |
| 5 | 0.1830 | 0.1018 | 0.0898 | 0.0891 | 0.1013 | 0.0876 | 0.0939 | 0.0070 |
| 6 | 0.1720 | 0.0826 | 0.0971 | 0.0841 | 0.0494 | 0.0770 | 0.0780 | 0.0176 |
| 7 | 0.1191 | 0.0419 | 0.0276 | 0.0106 | 0.0158 | 0.0466 | 0.0285 | 0.0157 |
| 8 | 0.1632 | 0.1027 | 0.1031 | 0.0825 | 0.0452 | 0.0815 | 0.0830 | 0.0236 |
| 9 | 0.1281 | 0.0589 | 0.0529 | 0.0500 | 0.0557 | 0.0465 | 0.0528 | 0.0048 |
| 10 | 0.1551 | 0.1008 | 0.0929 | 0.0892 | 0.0976 | 0.0797 | 0.0921 | 0.0082 |
| 11 | 0.2753 | 0.1097 | 0.0915 | 0.0962 | 0.0710 | 0.0415 | 0.0820 | 0.0266 |
| 12 | 0.3617 | 0.1344 | 0.0810 | 0.1001 | 0.1395 | 0.1462 | 0.1202 | 0.0282 |
| 13 | 1.5975 | 0.7856 | 0.7394 | 0.8523 | 0.9314 | 0.8181 | 0.8254 | 0.0724 |
| 14 | 1.3810 | 0.6781 | 0.5364 | 0.5951 | 0.6030 | 0.5379 | 0.5901 | 0.0582 |
| 15 | 1.5720 | 0.8810 | 0.7233 | 0.8469 | 0.7539 | 0.7606 | 0.7932 | 0.0673 |
| 16 | 1.7327 | 0.7280 | 0.7647 | 0.7178 | 0.6973 | 0.7043 | 0.7224 | 0.0265 |
| 17 | 2.0729 | 0.5362 | 0.5050 | 0.5162 | 0.5272 | 0.5348 | 0.5239 | 0.0132 |
| 18 | 1.0352 | 0.4492 | 0.4113 | 0.3349 | 0.4464 | 0.4670 | 0.4217 | 0.0526 |
| 19 | 1.0632 | 0.4363 | 0.4082 | 0.3990 | 0.4183 | 0.3424 | 0.4008 | 0.0355 |
| 20 | 0.2779 | 0.1175 | 0.1192 | 0.0811 | 0.0658 | 0.1027 | 0.0973 | 0.0233 |
| 21 | 1.4188 | 0.4487 | 0.4099 | 0.4271 | 0.4227 | 0.3804 | 0.4178 | 0.0251 |
| 22 | 1.5550 | 0.6988 | 0.6640 | 0.6351 | 0.6352 | 0.6124 | 0.6491 | 0.0333 |
| 23 | 0.4094 | 0.2231 | 0.2257 | 0.1481 | 0.2318 | 0.1662 | 0.1990 | 0.0388 |
| 24 | 1.8527 | 0.7899 | 0.7797 | 1.1874 | 0.6670 | 0.5592 | 0.7966 | 0.2378 |
| 25 | 1.5412 | 0.5734 | 0.6171 | 0.5745 | 0.5765 | 0.3674 | 0.5418 | 0.0992 |
| 26 | 2.0028 | 0.5773 | 0.6031 | 0.5869 | 0.6206 | 0.3548 | 0.5485 | 0.1096 |
| 27 | 1.5220 | 0.9613 | 0.8666 | 0.8123 | 0.9015 | 0.6986 | 0.8481 | 0.0995 |
| 28 | 1.7561 | 0.7851 | 0.7750 | 0.7586 | 0.8168 | 0.4322 | 0.7135 | 0.1587 |

| | | | | | | | | |
|---|---|---|---|---|---|---|---|---|
| 29 | 1.7414 | 0.7286 | 0.7135 | 0.6734 | 0.6403 | 0.4883 | 0.6488 | 0.0962 |
| 30 | 1.6018 | 0.6129 | 0.6264 | 0.6330 | 0.6408 | 0.4361 | 0.5899 | 0.0866 |

| | | Water Method | | | | | | |
|---|---|---|---|---|---|---|---|---|
| specimens | dry weight (g) | RM 1 | RM 2 | RM 3 | RM 4 | RM 5 | Mean | SD |
| 1 | 0.0554 | 0.0106 | 0.0108 | 0.0054 | 0.0250 | 0.0142 | 0.0132 | 0.0073 |
| 2 | 0.0377 | 0.0113 | 0.0080 | 0.0094 | 0.0110 | 0.0073 | 0.0094 | 0.0018 |
| 3 | 0.2311 | 0.0899 | 0.0635 | 0.0626 | 0.0872 | 0.1029 | 0.0812 | 0.0176 |
| 4 | 0.0827 | 0.0308 | 0.0358 | 0.0332 | 0.0440 | 0.0226 | 0.0333 | 0.0078 |
| 5 | 0.1830 | 0.0901 | 0.0627 | 0.1010 | 0.1421 | 0.1090 | 0.1010 | 0.0289 |
| 6 | 0.1720 | 0.0676 | 0.0554 | 0.0609 | 0.0834 | 0.0600 | 0.0655 | 0.0109 |
| 7 | 0.1191 | 0.0215 | 0.0315 | 0.0285 | 0.0403 | 0.0481 | 0.0340 | 0.0104 |
| 8 | 0.1632 | 0.0787 | 0.0777 | 0.0745 | 0.0839 | 0.0840 | 0.0798 | 0.0041 |
| 9 | 0.1281 | 0.0551 | 0.0370 | 0.0399 | 0.0468 | 0.0392 | 0.0436 | 0.0074 |
| 10 | 0.1551 | 0.1060 | 0.0596 | 0.0588 | 0.0700 | 0.0841 | 0.0757 | 0.0198 |
| 11 | 0.2753 | 0.0902 | 0.1085 | 0.0988 | 0.0994 | 0.1084 | 0.1011 | 0.0077 |
| 12 | 0.3617 | 0.1503 | 0.1568 | 0.1486 | 0.1172 | 0.1265 | 0.1399 | 0.0171 |
| 13 | 1.5975 | 0.7709 | 1.0312 | 0.8658 | 0.8956 | 0.9079 | 0.8943 | 0.0935 |
| 14 | 1.3810 | 0.6112 | 0.7168 | 0.7133 | 0.6973 | 0.6824 | 0.6842 | 0.0431 |
| 15 | 1.5720 | 0.8170 | 0.7602 | 0.7981 | 0.9895 | 1.1685 | 0.9067 | 0.1708 |
| 16 | 1.7327 | 0.7320 | 0.7480 | 0.6652 | 0.8382 | 0.5462 | 0.7059 | 0.1085 |
| 17 | 2.0729 | 0.4089 | 0.4868 | 0.4144 | 0.5354 | 0.3985 | 0.4488 | 0.0597 |
| 18 | 1.0352 | 0.5078 | 0.4869 | 0.5223 | 0.5420 | 0.5148 | 0.5148 | 0.0201 |
| 19 | 1.0632 | 0.2990 | 0.3949 | 0.4310 | 0.2731 | 0.3551 | 0.3506 | 0.0654 |
| 20 | 0.2779 | 0.1177 | 0.0728 | 0.1105 | 0.1365 | 0.0630 | 0.1001 | 0.0311 |
| 21 | 1.4188 | 0.2781 | 0.3291 | 0.4719 | 0.4996 | 0.4881 | 0.4134 | 0.1023 |
| 22 | 1.5550 | 0.5719 | 0.7729 | 0.7147 | 0.5839 | 0.8260 | 0.6939 | 0.1130 |
| 23 | 0.4094 | 0.1683 | 0.2488 | 0.2597 | 0.2693 | 0.2445 | 0.2381 | 0.0402 |
| 24 | 1.8527 | 0.7001 | 0.8260 | 0.6703 | 0.5908 | 0.7688 | 0.7112 | 0.0905 |
| 25 | 1.5412 | 0.4525 | 0.5755 | 0.6031 | 0.4641 | 0.2834 | 0.4757 | 0.1263 |
| 26 | 2.0028 | 0.6519 | 0.6144 | 0.6190 | 0.7020 | 0.3627 | 0.5900 | 0.1318 |
| 27 | 1.5220 | 0.9456 | 0.9458 | 1.0601 | 1.0614 | 0.9409 | 0.9908 | 0.0639 |
| 28 | 1.7561 | 0.8185 | 0.9748 | 0.9020 | 0.7917 | 0.6796 | 0.8333 | 0.1122 |
| 29 | 1.7414 | 0.6690 | 0.6943 | 0.6536 | 0.8620 | 0.5829 | 0.6924 | 0.1035 |
| 30 | 1.6018 | 0.5660 | 0.6057 | 0.6630 | 0.5381 | 0.5670 | 0.5880 | 0.0484 |

| | | CT method | |
|---|---|---|---|
| | | RM 1 | |
| specimens | dry weight (g) | Mean | SD |
| 1 | 0.0554 | 0.0031 | 0.0006 |
| 2 | 0.0377 | 0.0001 | 0.0000 |
| 3 | 0.2311 | 0.0521 | 0.0057 |
| 4 | 0.0827 | 0.0088 | 0.0013 |
| 5 | 0.1830 | 0.0530 | 0.0056 |
| 6 | 0.1720 | 0.0419 | 0.0047 |
| 7 | 0.1191 | 0.0204 | 0.0027 |
| 8 | 0.1632 | 0.0419 | 0.0046 |

| | | | |
|---|---|---|---|
| 9 | 0.1281 | 0.0210 | 0.0029 |
| 10 | 0.1551 | 0.0464 | 0.0050 |
| 11 | 0.2753 | 0.0469 | 0.0053 |
| 12 | 0.3617 | 0.0511 | 0.0053 |
| 13 | 1.5975 | 0.6291 | 0.0330 |
| 14 | 1.3810 | 0.4019 | 0.0290 |
| 15 | 1.5720 | 0.5044 | 0.0335 |
| 16 | 1.7327 | 0.4868 | 0.0612 |
| 17 | 2.0729 | 0.2979 | 0.0432 |
| 18 | 1.0352 | 0.2503 | 0.0312 |
| 19 | 1.0632 | 0.2177 | 0.0189 |
| 20 | 0.2779 | 0.0477 | 0.0057 |
| 21 | 1.4188 | 0.2261 | 0.0191 |
| 22 | 1.5550 | 0.4107 | 0.0294 |
| 23 | 0.4094 | 0.0978 | 0.0093 |
| 24 | 1.8527 | 0.5592 | 0.0660 |
| 25 | 1.5412 | 0.3674 | 0.0485 |
| 26 | 2.0028 | 0.3548 | 0.0482 |
| 27 | 1.5220 | 0.6986 | 0.0668 |
| 28 | 1.7561 | 0.4322 | 0.0546 |
| 29 | 1.7414 | 0.4883 | 0.0546 |
| 30 | 1.6018 | 0.4361 | 0.0550 |

*Tegula viridula*

| | | **Volume estimates (cm³) - Repeated measures (RM)** | | | | | | |
|---|---|---|---|---|---|---|---|---|
| | | Sand Method | | | | | | |
| specimens | dry weight (g) | RM 1 | RM 2 | RM 3 | RM 4 | RM 5 | Mean | SD |
| 1 | 3.7532 | 1.0473 | 1.2323 | 0.9714 | 1.0092 | 0.9177 | 1.0356 | 0.1199 |
| 2 | 2.0491 | 0.5922 | 0.5378 | 0.5469 | 0.5673 | 0.4680 | 0.5424 | 0.0466 |
| 3 | 1.1965 | 0.5533 | 0.5638 | 0.5145 | 0.4970 | 0.4200 | 0.5097 | 0.0572 |
| 4 | 2.3702 | 0.6381 | 0.6298 | 0.6918 | 0.6644 | 0.6328 | 0.6514 | 0.0264 |
| 5 | 0.1272 | 0.0315 | 0.0396 | 0.0331 | 0.0375 | 0.0271 | 0.0338 | 0.0049 |
| 6 | 0.1280 | 0.0451 | 0.0539 | 0.0451 | 0.0415 | 0.0328 | 0.0437 | 0.0076 |
| 7 | 0.3905 | 0.1385 | 0.1094 | 0.1315 | 0.1237 | 0.1129 | 0.1232 | 0.0123 |
| 8 | 1.9302 | 0.6301 | 0.6430 | 0.6199 | 0.5854 | 0.5132 | 0.5983 | 0.0522 |
| 9 | 1.6565 | 0.4914 | 0.5433 | 0.5216 | 0.5196 | 0.4080 | 0.4968 | 0.0529 |
| 10 | 2.1020 | 0.5248 | 0.2275 | 0.5816 | 0.5680 | 0.5165 | 0.4837 | 0.1458 |
| 11 | 0.1578 | 0.0471 | 0.0479 | 0.0354 | 0.0374 | 0.0356 | 0.0407 | 0.0063 |
| 12 | 0.7212 | 0.1993 | 0.1899 | 0.1810 | 0.1985 | 0.1562 | 0.1850 | 0.0177 |
| 13 | 1.0712 | 0.2934 | 0.2696 | 0.2609 | 0.2611 | 0.2666 | 0.2703 | 0.0134 |
| 14 | 2.1467 | 0.6028 | 0.5772 | 0.5461 | 0.5375 | 0.4920 | 0.5511 | 0.0420 |
| 15 | 1.3983 | 0.3389 | 0.3899 | 0.3433 | 0.3440 | 0.3285 | 0.3489 | 0.0237 |
| 16 | 0.8106 | 0.3485 | 0.3370 | 0.3004 | 0.3405 | 0.3352 | 0.3323 | 0.0186 |
| 17 | 2.2913 | 0.6573 | 0.7117 | 0.6423 | 0.7007 | 0.6576 | 0.6739 | 0.0304 |
| 18 | 1.3071 | 0.3426 | 0.3743 | 0.3392 | 0.3753 | 0.3701 | 0.3603 | 0.0179 |
| 19 | 0.3841 | 0.0980 | 0.0918 | 0.0961 | 0.1003 | 0.0854 | 0.0943 | 0.0059 |

| | | | | | | | | |
|---|---|---|---|---|---|---|---|---|
| 20 | 1.4843 | 0.5190 | 0.5249 | 0.5270 | 0.5564 | 0.4809 | 0.5216 | 0.0270 |
| 21 | 5.6199 | 2.2940 | 2.1392 | 2.1269 | 2.1026 | 1.7968 | 2.0919 | 0.1813 |
| 22 | 4.4137 | 1.3573 | 1.3177 | 1.2651 | 1.3797 | 1.1325 | 1.2905 | 0.0984 |
| 23 | 4.2301 | 1.4469 | 1.4591 | 1.2474 | 1.4587 | 1.0404 | 1.3305 | 0.1855 |
| 24 | 4.2505 | 1.2109 | 1.2143 | 1.1906 | 1.1909 | 0.8938 | 1.1401 | 0.1381 |
| 25 | 3.8610 | 1.1863 | 1.2560 | 1.3011 | 1.2635 | 0.8470 | 1.1708 | 0.1857 |
| 26 | 4.5109 | 1.4196 | 1.7185 | 1.5462 | 1.4615 | 1.2400 | 1.4772 | 0.1752 |
| 27 | 3.8023 | 1.3505 | 1.4485 | 1.6241 | 1.4791 | 1.2692 | 1.4343 | 0.1346 |
| 28 | 3.7447 | 1.2586 | 1.1735 | 1.2916 | 1.2663 | 1.1510 | 1.2282 | 0.0619 |
| 29 | 3.5470 | 1.2642 | 1.2971 | 1.2801 | 1.2172 | 1.2452 | 1.2608 | 0.0310 |
| 30 | 2.0625 | 0.6250 | 0.5091 | 0.6120 | 0.6145 | 0.4385 | 0.5598 | 0.0825 |

| | | Water Method | | | | | | |
|---|---|---|---|---|---|---|---|---|
| specimens | dry weight (g) | RM 1 | RM 2 | RM 3 | RM 4 | RM 5 | Mean | SD |
| 1 | 3.7532 | 1.0095 | 1.2434 | 1.0080 | 1.1330 | 0.9488 | 1.0685 | 0.1186 |
| 2 | 2.0491 | 0.6239 | 0.6294 | 0.4712 | 0.5836 | 0.6105 | 0.5837 | 0.0653 |
| 3 | 1.1965 | 0.6314 | 0.6591 | 0.6224 | 0.5509 | 0.5671 | 0.6062 | 0.0455 |
| 4 | 2.3702 | 0.7642 | 0.5766 | 0.6503 | 0.7069 | 0.5950 | 0.6586 | 0.0780 |
| 5 | 0.1272 | 0.0241 | 0.0224 | 0.0259 | 0.0266 | 0.0161 | 0.0230 | 0.0042 |
| 6 | 0.1280 | 0.0202 | 0.0390 | 0.0405 | 0.0431 | 0.0513 | 0.0388 | 0.0114 |
| 7 | 0.3905 | 0.1094 | 0.1137 | 0.1167 | 0.0931 | 0.0887 | 0.1043 | 0.0126 |
| 8 | 1.9302 | 0.6039 | 0.5566 | 0.6571 | 0.7463 | 0.7279 | 0.6584 | 0.0805 |
| 9 | 1.6565 | 0.5341 | 0.5770 | 0.4740 | 0.4296 | 0.5486 | 0.5127 | 0.0598 |
| 10 | 2.1020 | 0.6393 | 0.6630 | 0.6386 | 0.7079 | 0.6512 | 0.6600 | 0.0286 |
| 11 | 0.1578 | 0.0500 | 0.0344 | 0.0396 | 0.0341 | 0.0173 | 0.0351 | 0.0118 |
| 12 | 0.7212 | 0.1932 | 0.1335 | 0.1688 | 0.1324 | 0.1502 | 0.1556 | 0.0257 |
| 13 | 1.0712 | 0.2919 | 0.3283 | 0.2858 | 0.2408 | 0.2637 | 0.2821 | 0.0327 |
| 14 | 2.1467 | 0.6206 | 0.5202 | 0.5295 | 0.5333 | 0.5817 | 0.5571 | 0.0428 |
| 15 | 1.3983 | 0.3569 | 0.3611 | 0.4255 | 0.2983 | 0.3834 | 0.3650 | 0.0462 |
| 16 | 0.8106 | 0.3534 | 0.3067 | 0.2812 | 0.3292 | 0.3327 | 0.3206 | 0.0276 |
| 17 | 2.2913 | 0.6884 | 0.8104 | 0.7465 | 0.7449 | 0.8584 | 0.7697 | 0.0657 |
| 18 | 1.3071 | 0.4370 | 0.4444 | 0.3587 | 0.3181 | 0.4793 | 0.4075 | 0.0666 |
| 19 | 0.3841 | 0.0745 | 0.0936 | 0.0845 | 0.0766 | 0.0693 | 0.0797 | 0.0095 |
| 20 | 1.4843 | 0.5990 | 0.5495 | 0.4616 | 0.5429 | 0.6235 | 0.5553 | 0.0623 |
| 21 | 5.6199 | 2.1616 | 2.3254 | 2.1085 | 2.1984 | 2.0677 | 2.1723 | 0.0991 |
| 22 | 4.4137 | 1.2443 | 1.3321 | 1.3252 | 1.3297 | 1.1625 | 1.2788 | 0.0747 |
| 23 | 4.2301 | 1.5095 | 1.3547 | 1.4475 | 1.4970 | 1.3945 | 1.4406 | 0.0661 |
| 24 | 4.2505 | 1.2274 | 1.2222 | 1.1790 | 1.0981 | 1.2225 | 1.1898 | 0.0549 |
| 25 | 3.8610 | 1.3386 | 1.0378 | 1.1884 | 1.2645 | 0.7489 | 1.1156 | 0.2333 |
| 26 | 4.5109 | 1.7446 | 1.6438 | 1.3705 | 1.6637 | 1.3421 | 1.5529 | 0.1837 |
| 27 | 3.8023 | 1.6227 | 1.4634 | 1.4762 | 1.5592 | 1.4483 | 1.5140 | 0.0744 |
| 28 | 3.7447 | 1.2235 | 1.0391 | 1.1865 | 1.2162 | 1.1346 | 1.1600 | 0.0761 |
| 29 | 3.5470 | 1.1709 | 1.1187 | 1.1049 | 1.2050 | 1.2171 | 1.1633 | 0.0502 |
| 30 | 2.0625 | 0.6268 | 0.5299 | 0.5701 | 0.5531 | 0.4802 | 0.5520 | 0.0538 |

| specimens | dry weight (g) | CT method | |
|---|---|---|---|
| | | RM 1 | |
| | | Mean | SD |
| 1 | 3.7532 | 0.8315 | 0.0320 |
| 2 | 2.0491 | 0.3632 | 0.0169 |
| 3 | 1.1965 | 0.3662 | 0.0172 |
| 4 | 2.3702 | 0.4459 | 0.0456 |
| 5 | 0.1272 | 0.0012 | 0.0003 |
| 6 | 0.1280 | 0.0105 | 0.0013 |
| 7 | 0.3905 | 0.0551 | 0.0042 |
| 8 | 1.9302 | 0.4405 | 0.0199 |
| 9 | 1.6565 | 0.3096 | 0.0155 |
| 10 | 2.1020 | 0.3997 | 0.0180 |
| 11 | 0.1578 | 0.0042 | 0.0007 |
| 12 | 0.7212 | 0.0640 | 0.0052 |
| 13 | 1.0712 | 0.1072 | 0.0076 |
| 14 | 2.1467 | 0.3860 | 0.0408 |
| 15 | 1.3983 | 0.1900 | 0.0114 |
| 16 | 0.8106 | 0.1625 | 0.0100 |
| 17 | 2.2913 | 0.4280 | 0.0211 |
| 18 | 1.3071 | 0.1757 | 0.0104 |
| 19 | 0.3841 | 0.0206 | 0.0022 |
| 20 | 1.4843 | 0.2758 | 0.0144 |
| 21 | 5.6199 | 1.6445 | 0.1210 |
| 22 | 4.4137 | 1.0585 | 0.0873 |
| 23 | 4.2301 | 1.0730 | 0.0869 |
| 24 | 4.2505 | 0.8449 | 0.0740 |
| 25 | 3.8610 | 0.8929 | 0.0771 |
| 26 | 4.5109 | 1.1998 | 0.0962 |
| 27 | 3.8023 | 1.1783 | 0.0942 |
| 28 | 3.7447 | 0.9264 | 0.0795 |
| 29 | 3.5470 | 0.9026 | 0.0779 |
| 30 | 2.0625 | 0.3614 | 0.0391 |

## Approach 4

*C. senegalensis*

| Method | specimen | RM 1 | RM 2 | RM 3 | RM 4 | RM 5 | Mean | SD |
|---|---|---|---|---|---|---|---|---|
| Sand | 1 | 4.6478 | 5.1032 | 5.0666 | 4.4787 | 4.8638 | 4.8320 | 0.2685 |
| | 2 | 5.9587 | 5.8568 | 6.3024 | 5.7987 | 6.4820 | 6.0797 | 0.2978 |
| | 3 | 4.3488 | 4.6228 | 4.8583 | 4.4444 | 5.7459 | 4.8041 | 0.5611 |
| Water | 1 | 5.7419 | 5.6684 | 5.4196 | 5.3913 | 5.2390 | 5.4920 | 0.2079 |
| | 2 | 6.8036 | 6.7052 | 6.7578 | 6.8106 | 5.9807 | 6.6116 | 0.3552 |

| | | | | | | | | | |
|---|---|---|---|---|---|---|---|---|---|
| | 3 | | 5.6204 | 5.5144 | 5.5826 | 5.5339 | 5.9090 | 5.6321 | 0.1603 |
| CT | 1 | Mean | 4.7910 | 5.1000 | 4.8670 | 6.0000 | 4.9030 | 5.1322 | 0.4983 |
| | | SD | 0.2360 | 0.2000 | 0.2240 | 0.3000 | 0.2600 | | |
| | 2 | Mean | 6.0000 | 6.1000 | 5.9000 | 6.0000 | 5.8000 | 5.9600 | 0.1140 |
| | | SD | 0.3000 | 0.3000 | 0.2000 | 0.2000 | 0.3000 | | |
| | 3 | Mean | 4.7490 | 4.8680 | 4.8220 | 4.8550 | 4.7220 | 4.8032 | 0.0647 |
| | | SD | 0.2210 | 0.2180 | 0.2110 | 0.1570 | 0.2400 | | |

*C. parthenopeum*

| Method | specimen | | RM 1 | RM 2 | RM 3 | RM 4 | RM 5 | Mean | SD |
|---|---|---|---|---|---|---|---|---|---|
| Sand | 1 | | 7.3053 | 6.4784 | 6.4775 | 7.8621 | 6.3235 | 6.8894 | 0.6667 |
| | 2 | | 8.3765 | 8.1727 | 8.1312 | 8.6998 | 8.2157 | 8.3192 | 0.2322 |
| | 3 | | 5.9972 | 5.0960 | 5.5381 | 5.9033 | 6.6939 | 5.8457 | 0.5921 |
| Water | 1 | | 7.3466 | 8.5104 | 7.6775 | 8.6504 | 8.5580 | 8.1486 | 0.5949 |
| | 2 | | 9.2144 | 9.4202 | 9.2920 | 9.4597 | 9.5008 | 9.3774 | 0.1201 |
| | 3 | | 8.5897 | 7.2313 | 8.3024 | 8.4711 | 7.1950 | 7.9579 | 0.6876 |
| CT | 1 | Mean | 7.5000 | 7.9000 | 7.4000 | 7.9000 | 7.4000 | 7.6200 | 0.2588 |
| | | SD | 0.4000 | 0.4000 | 0.3000 | 0.3000 | 0.4000 | | |
| | 2 | Mean | 8.6000 | 8.5000 | 8.5000 | 8.6000 | 8.4000 | 8.5200 | 0.0837 |
| | | SD | 0.4000 | 0.3000 | 0.3000 | 0.2000 | 0.4000 | | |
| | 3 | Mean | 7.3000 | 7.9000 | 7.5000 | 7.7000 | 7.3000 | 7.5400 | 0.2608 |
| | | SD | 0.4000 | 0.4000 | 0.4000 | 0.3000 | 0.4000 | | |

*S. haemastoma*

| Method | specimen | | RM 1 | RM 2 | RM 3 | RM 4 | RM 5 | Mean | SD |
|---|---|---|---|---|---|---|---|---|---|
| Sand | 1 | | 6.1481 | 6.4026 | 6.3194 | 6.1765 | 6.2597 | 6.2613 | 0.1041 |
| | 2 | | 8.4713 | 8.8092 | 8.1769 | 8.5023 | 8.1896 | 8.4299 | 0.2610 |
| | 3 | | 6.5258 | 6.5977 | 6.4616 | 6.7656 | 6.5599 | 6.5821 | 0.1141 |
| Water | 1 | | 6.9333 | 6.6268 | 6.6190 | 6.7777 | 6.4151 | 6.6744 | 0.1938 |
| | 2 | | 8.4775 | 8.5280 | 9.1600 | 8.7166 | 9.4353 | 8.8635 | 0.4177 |
| | 3 | | 6.9552 | 6.9019 | 7.1881 | 7.1080 | 9.6426 | 7.5592 | 1.1703 |
| CT | 1 | Mean | 6.2000 | 6.3000 | 6.0000 | 6.5000 | 6.3000 | 6.2600 | 0.1817 |
| | | SD | 0.2000 | 0.3000 | 0.3000 | 0.2000 | 0.3000 | | |
| | 2 | Mean | 8.0000 | 8.3000 | 8.2000 | 9.5000 | 8.3000 | 8.4600 | 0.5941 |
| | | SD | 0.2000 | 0.3000 | 0.3000 | 0.3000 | 0.4000 | | |
| | 3 | Mean | 6.4000 | 7.1000 | 6.4000 | 6.8000 | 7.8000 | 6.9000 | 0.5831 |
| | | SD | 0.2000 | 0.3000 | 0.3000 | 0.2000 | 0.5000 | | |

*C. atratum*_Large

| Method | specimen | RM 1 | RM 2 | RM 3 | RM 4 | RM 5 | Mean | SD |
|---|---|---|---|---|---|---|---|---|
| Sand | 1 | 0.7899 | 0.7797 | 1.1874 | 0.6670 | 0.5592 | 0.7966 | 0.2378 |
| | 2 | 0.5734 | 0.6171 | 0.5745 | 0.5765 | 0.3674 | 0.5418 | 0.0992 |
| | 3 | 0.6129 | 0.6264 | 0.6330 | 0.6408 | 0.4361 | 0.5899 | 0.0866 |
| Water | 1 | 0.7001 | 0.8260 | 0.6703 | 0.5908 | 0.7688 | 0.7112 | 0.0905 |
| | 2 | 0.4525 | 0.5755 | 0.6031 | 0.4641 | 0.2834 | 0.4757 | 0.1263 |

| | | | | | | | | | |
|---|---|---|---|---|---|---|---|---|---|
| | 3 | | 0.5660 | 0.6057 | 0.6630 | 0.5381 | 0.5670 | 0.5880 | 0.0484 |
| CT | 1 | Mean | 0.6500 | 0.6320 | 0.6450 | 0.6130 | | 0.6350 | 0.0165 |
| | | SD | 0.0510 | 0.0370 | 0.0380 | 0.0450 | | | |
| | 2 | Mean | 0.4630 | 0.4550 | 0.4560 | 0.4430 | 0.4670 | 0.4568 | 0.0092 |
| | | SD | 0.0410 | 0.0300 | 0.0270 | 0.0360 | 0.0570 | | |
| | 3 | Mean | 0.5230 | 0.5330 | 0.5350 | 0.5380 | | 0.5323 | 0.0065 |
| | | SD | 0.0440 | 0.0330 | 0.0310 | 0.0410 | | | |

*C. atratum*_Small

| Method | specimen | | RM 1 | RM 2 | RM 3 | RM 4 | RM 5 | Mean | SD |
|---|---|---|---|---|---|---|---|---|---|
| Sand | 1 | | 0.1101 | 0.1121 | 0.1138 | 0.1125 | 0.1235 | 0.1144 | 0.0053 |
| | 2 | | 0.1218 | 0.1266 | 0.1265 | 0.1227 | 0.1189 | 0.1380 | 0.0133 |
| | 3 | | 0.1027 | 0.1031 | 0.0825 | 0.0452 | 0.0815 | 0.0830 | 0.0236 |
| Water | 1 | | 0.1264 | 0.1401 | 0.1336 | 0.1600 | 0.1301 | 0.1380 | 0.0133 |
| | 2 | | 0.1424 | 0.1399 | 0.1586 | 0.1467 | 0.1452 | 0.1465 | 0.0072 |
| | 3 | | 0.0787 | 0.0777 | 0.0745 | 0.0839 | 0.0840 | 0.0798 | 0.0041 |
| CT | 1 | Mean | 0.0650 | 0.0670 | 0.0640 | 0.0860 | 0.0640 | 0.0692 | 0.0095 |
| | | SD | 0.0090 | 0.0070 | 0.0060 | 0.0130 | 0.0120 | | |
| | 2 | Mean | 0.0720 | 0.0740 | 0.0730 | 0.0740 | 0.0770 | 0.0740 | 0.0019 |
| | | SD | 0.0100 | 0.0080 | 0.0070 | 0.0100 | 0.0140 | | |
| | 3 | Mean | 0.0570 | 0.0680 | 0.0570 | 0.0590 | | 0.0603 | 0.0053 |
| | | SD | 0.0080 | 0.0060 | 0.0060 | 0.0110 | | | |

*T. viridula*_Large

| Method | specimen | | RM 1 | RM 2 | RM 3 | RM 4 | RM 5 | Mean | SD |
|---|---|---|---|---|---|---|---|---|---|
| Sand | 1 | | 1.3520 | 1.2822 | 1.1114 | 1.2683 | 0.9695 | 1.1967 | 0.1545 |
| | 2 | | 1.0473 | 1.2323 | 0.9714 | 1.0092 | 0.9177 | 1.0356 | 0.1199 |
| | 3 | | 1.4289 | 1.4879 | 1.4707 | 1.4362 | 1.1961 | 1.4040 | 0.1187 |
| Water | 1 | | 1.1916 | 1.2596 | 1.2267 | 1.4198 | 1.0410 | 1.2277 | 0.1361 |
| | 2 | | 1.0095 | 1.2434 | 1.0080 | 1.1330 | 0.9488 | 1.0685 | 0.1186 |
| | 3 | | 1.4568 | 1.3882 | 1.5252 | 1.5156 | 1.3901 | 1.4552 | 0.0657 |
| CT | 1 | Mean | 1.1790 | 1.2830 | 1.1430 | 1.0730 | 1.1220 | 1.1600 | 0.0787 |
| | | SD | 0.0690 | 0.0530 | 0.0440 | 0.0060 | 0.1000 | | |
| | 2 | Mean | 0.9060 | 0.9490 | 0.9900 | 0.9340 | 0.9330 | 0.9424 | 0.0308 |
| | | SD | 0.0570 | 0.0410 | 0.0360 | 0.0050 | 0.0850 | | |
| | 3 | Mean | 1.3810 | 1.6270 | 1.3850 | 1.3230 | 1.3280 | 1.4088 | 0.1253 |
| | | SD | 0.7840 | 0.0630 | 0.0510 | 0.0070 | 0.1110 | | |

*T. viridula*_Small

| Method | specimen | | RM 1 | RM 2 | RM 3 | RM 4 | RM 5 | Mean | SD |
|---|---|---|---|---|---|---|---|---|---|
| Sand | 1 | | 0.3485 | 0.3370 | 0.3004 | 0.3405 | 0.3352 | 0.3323 | 0.0186 |
| | 2 | | 0.2192 | 0.1673 | 0.1869 | 0.2097 | 0.2020 | 0.1970 | 0.0204 |
| | 3 | | 0.1359 | 0.1311 | 0.1495 | 0.1398 | 0.1464 | 0.1406 | 0.0075 |
| Water | 1 | | 0.3534 | 0.3067 | 0.2812 | 0.3292 | 0.3327 | 0.3206 | 0.0276 |
| | 2 | | 0.1938 | 0.1758 | 0.2027 | 0.2432 | 0.2263 | 0.2083 | 0.0266 |

| | | | | | | | | | |
|---|---|---|---|---|---|---|---|---|---|
| | 3 | | 0.1553 | 0.1603 | 0.1679 | 0.1537 | 0.1711 | 0.1617 | 0.0076 |
| CT | 1 | Mean | 0.2280 | 0.2280 | 0.2880 | 0.2360 | 0.2340 | 0.2428 | 0.0255 |
| | | SD | 0.0210 | 0.0150 | 0.0150 | 0.0020 | 0.0300 | | |
| | 2 | Mean | 0.1370 | 0.1430 | 0.1510 | 0.1620 | 0.1340 | 0.1454 | 0.0113 |
| | | SD | 0.0140 | 0.0110 | 0.0100 | 0.0140 | 0.0190 | | |
| | 3 | Mean | 0.1050 | 0.0960 | 0.1080 | 0.1110 | 0.1020 | 0.1044 | 0.0058 |
| | | SD | 0.0120 | 0.0080 | 0.0080 | 0.0110 | 0.0160 | | |
